# Diagnostic value of machine-learning using conventional magnetic resonance imaging markers for pediatric idiopathic intracranial hypertension: a retrospective study

**DOI:** 10.1007/s00247-026-06638-7

**Published:** 2026-05-23

**Authors:** Ferhat Cuce, Gokalp Tulum, Muhammet Ikbal Işık, Mehmet Baştemur, Mutluay Arslan, Bülent Ünay, Onur Osman

**Affiliations:** 1 Department of Radiology, Gulhane Training and Research Hospital, Sağlık Bilimleri Üniversitesi, Ankara, Turkey; 2 Department of Electrical and Electronics Engineering, Istanbul Topkapi University, Prof. Muammer Aksoy Cad. No: 10, Kazlıçeşme, Zeytinburnu, Istanbul, Türkiye; 3 Department of Radiology, Ağrı Training, and Research Hospital, Ağrı, Turkey; 4 Division of Child Neurology, Deparment of Pediatrics, Eskişehir City Hospital, Eskişehir, Turkey; 5 Research Hospital Division of Child Neurology, Gulhane Training, Sağlık Bilimleri Üniversitesi, Ankara, Turkey

**Keywords:** Adolescent, Child, Diagnosis, computer-assisted, Machine-learning algorithms, Magnetic resonance imaging, Optic nerve, Papilledema, Pseudotumor cerebri

## Abstract

**Background:**

Pediatric idiopathic intracranial hypertension can be challenging to diagnose; magnetic resonance imaging (MRI) signs are considered supportive, while lumbar puncture remains the diagnostic reference.

**Objective:**

We evaluated whether a compact set of conventional magnetic resonance imaging markers, analyzed with supervised machine-learning, can assist in the diagnosis of idiopathic intracranial hypertension in children presenting with headache.

**Materials and methods:**

In this retrospective single-center study, 62 pediatric patients with idiopathic intracranial hypertension and 62 headache controls without papilledema or structural pathology were analyzed. All lumbar puncture procedures in the idiopathic intracranial hypertension group were performed under benzodiazepine sedation. Twenty-four demographic and quantitative MRI features (optic nerve sheath and globe metrics, pituitary measurements, venous sinus findings, and posterior fossa measures) were extracted. Six classifiers (random forest, support vector machine, multilayer perceptron, XGBoost, *k*-nearest neighbors, and Bagging) were trained using repeated nested cross-validation with Bayesian hyperparameter optimization; performance was summarized on held-out folds.

**Results:**

Across models, accuracies were approximately 0.70–0.74, sensitivities approximately 0.64–0.72, and specificities approximately 0.69–0.84; among non–support vector machine models, the area under the receiver operating characteristic curve was approximately 0.80–0.82. Feature selection consistently retained anatomically plausible markers, led by optic nerve sheath measurements, posterior globe flattening, pituitary/sella configuration, and venous sinus abnormalities. The resulting models generate uncalibrated predicted probabilities for decision support rather than binary diagnostic labels. Performance was estimated across repeated validation splits.

**Conclusion:**

Machine-learning applied to routinely obtainable MRI markers yields moderate diagnostic performance for pediatric idiopathic intracranial hypertension. Given that lumbar puncture remains the gold standard, these models are intended as decision-support tools to complement clinical assessment.

**Graphical abstract:**

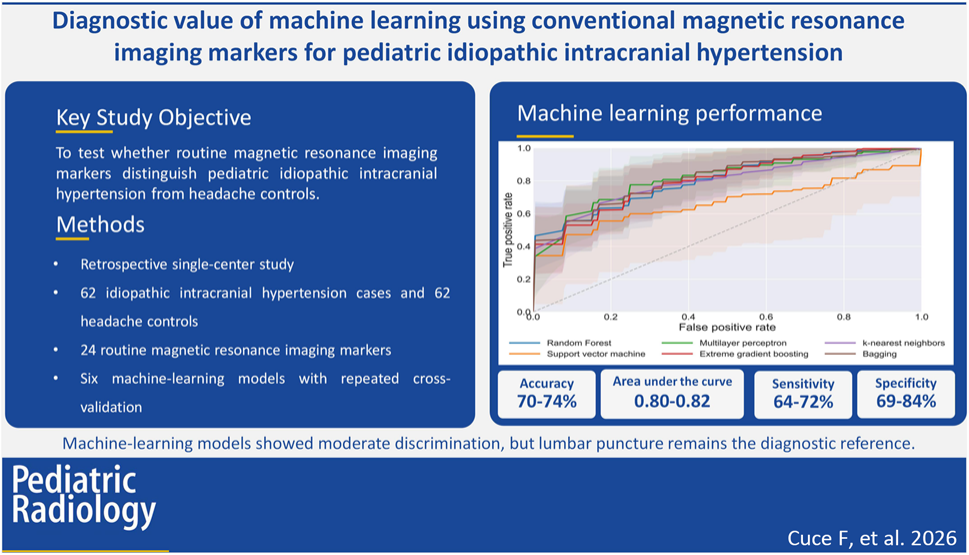

**Supplementary Information:**

The online version contains supplementary material available at 10.1007/s00247-026-06638-7.

## Introduction

Idiopathic intracranial hypertension, commonly known as pseudotumor cerebri, is a neurological disorder characterized by elevated intracranial pressure without an identifiable cause such as a brain tumor, infection, vascular lesion, or hydrocephalus [1]. Although more frequently diagnosed in obese women of childbearing age, idiopathic intracranial hypertension presents a distinct clinical challenge in children because of its different epidemiologic profile, variable presentation, and potential for significant morbidity.

The estimated annual incidence in childhood is between 0.5 and 1 per 100,000 children [2, 3]. In prepubertal children, idiopathic intracranial hypertension affects boys and girls similarly, whereas in adolescents a marked female predominance emerges, mirroring the adult pattern [2, 4]. Risk factors also change with age. Obesity is an important factor in adolescents but is less strongly associated in younger children [4, 5]. In younger patients, secondary causes and associated conditions should be considered carefully, including exposure to certain medications (e.g., tetracycline antibiotics, vitamin A derivatives, and corticosteroid withdrawal), underlying endocrine disorders, and cerebral venous sinus thrombosis [2].

The diagnosis of pediatric idiopathic intracranial hypertension is based on the modified Dandy criteria, which require the presence of signs and symptoms of increased intracranial pressure (such as headache or papilledema), documented elevated cerebrospinal fluid (CSF) opening pressure on lumbar puncture, normal CSF composition, and exclusion of other causes by normal neuroimaging [1, 6]. Diagnosis in children can nevertheless be challenging. Younger children may present with nonspecific symptoms such as irritability, lethargy, or sixth nerve palsy rather than classic headache, and the absence of papilledema does not exclude the condition, especially in infants with open cranial sutures [2].

The major complication of idiopathic intracranial hypertension is permanent and potentially severe vision loss due to chronic papilledema. If increased intracranial pressure is not recognized and treated adequately, irreversible optic nerve atrophy may develop [7]. Therefore, a high index of suspicion, prompt ophthalmologic evaluation, and a multidisciplinary treatment approach are essential to preserve vision and improve long-term outcomes in affected children [8].

In a child with suspected idiopathic intracranial hypertension, brain magnetic resonance imaging (MRI) is an essential step in the diagnostic work-up. Its primary role is to exclude other causes of increased intracranial pressure rather than to establish the diagnosis of idiopathic intracranial hypertension. A secondary role is to identify structural imaging findings that support chronically elevated intracranial pressure.

Although not diagnostic on their own, certain MRI findings are strongly associated with chronically elevated intracranial pressure. These findings include empty sella, perioptic subarachnoid space widening, tortuosity of the optic nerve orbital segment, flattening of the posterior contour of the ocular globes, slit-like ventricles, and transverse venous sinus stenosis with external compression. Venous sinus stenosis has been reported frequently in idiopathic intracranial hypertension and has been characterized morphologically in MR studies [9].

MRI findings supportive of idiopathic intracranial hypertension can also be observed in individuals without the condition, which may lead to false-positive interpretations when they are considered in isolation [10]. In a systematic review and meta-analysis of case–control studies, individual MRI signs, including empty sella and posterior globe flattening, showed generally high pooled specificity but heterogeneous and often limited pooled sensitivity (pooled sensitivity range 6.1–68.6%; pooled specificity range 84.0–99.2%) [10]. In a case–control cohort, posterior globe flattening was present in 65.9% of patients with idiopathic intracranial hypertension versus 4.5% of controls (estimated sensitivity ~65.9% and specificity ~95.5%) [11]. In the same cohort, optic nerve sheath distension (52.3% vs. 11.4%) and optic nerve tortuosity (54.5% vs. 9.1%) were also more common in idiopathic intracranial hypertension than in controls, supporting their value within a multiparametric assessment rather than as stand-alone criteria [11].

Several adult studies of idiopathic intracranial hypertension have sought to make MRI assessment more objective by quantifying imaging markers and evaluating diagnostic accuracy using reproducible readouts. Adult cohorts have examined the diagnostic accuracy of conventional brain MRI and magnetic resonance venography (MRV) sign combinations in suspected idiopathic intracranial hypertension [12], volumetric measurements of the optic nerve sheath and pituitary for diagnosis and post-treatment follow-up [13], and practical thresholds for routinely measured MRI parameters [14, 15]. More advanced approaches, including MR elastography [16], automated image analysis of posterior scleral flattening and optic nerve head protrusion [17], and multivariable or machine-learning–based models combining multiple MRI descriptors [18, 19], have also been reported.

However, direct translation of adult-derived quantitative thresholds and model-based scores to children is challenging because normative morphometric baselines are age-dependent, measurements may vary between observers, and some pressure-related findings can also be present in healthy children. We hypothesized that a compact set of conventional MRI markers, analyzed jointly with supervised machine-learning, would discriminate pediatric idiopathic intracranial hypertension from headache controls. Accordingly, in this study we aimed to quantify the diagnostic value of MRI findings traditionally considered “supportive” for idiopathic intracranial hypertension in children by training and interpreting supervised machine-learning models to distinguish pediatric idiopathic intracranial hypertension from the control group.

## Materials and methods

### Patients

The local ethics committee approved this retrospective study (date: 27.11.2025, decision number: 470) and written consent was waived. This retrospective study included pediatric patients (<18 years of age) who were evaluated at the Department of Pediatric Neurology, University of Health Sciences Gülhane Training and Research Hospital between January 2020 and January 2025, and were diagnosed with idiopathic intracranial hypertension. Patients with an elevated lumbar puncture opening pressure (≥28 cm H_2_O in sedated and/or obese children, ≥25 cm H_2_O in non-sedated, non-obese children), normal CSF biochemical and cytological findings, and clinical features consistent with pseudotumor cerebri (PTC), such as papilledema, were included in the patient group. All patients had no mass lesions, hydrocephalus, venous sinus thrombosis, or other structural brain abnormalities on MRI. In our cohort, MRI was performed prior to lumbar puncture, with a median interval of approximately 1 week between magnetic resonance imaging and lumbar puncture, and no medical treatment directed at idiopathic intracranial hypertension was initiated before lumbar puncture. In our cohort, all lumbar punctures were performed under benzodiazepine sedation. Demographic and neuroimaging data were retrospectively collected using a standardized data collection form.

An equal number of children presenting with headache without papilledema on examination and showing no structural intracranial pathology on MRI were recruited as the control group. MRI signs supportive of idiopathic intracranial hypertension, such as empty sella or optic nerve sheath distension, were recorded for analysis but were not used for group assignment. Control patients had no clinical findings suggestive of raised intracranial pressure and no neuroimaging features requiring further evaluation for raised intracranial pressure; therefore, lumbar puncture was not clinically indicated and was not performed in this group. Patients with identified or clinically suspected secondary causes of intracranial hypertension, abnormal CSF findings suggestive of infection, inflammation, or neoplasia, or neuroimaging findings indicating hydrocephalus, mass lesion, or other structural brain abnormalities were excluded from the study.

In the idiopathic intracranial hypertension group, a standardized diagnostic approach was applied as part of routine clinical practice. Secondary causes of intracranial hypertension were assessed during the diagnostic work-up, including medication history and targeted evaluation for secondary etiologies, and patients with suspected secondary intracranial hypertension were excluded.

In the control group (headache patients), children were selected, given the retrospective design, from those in whom medication history had been routinely recorded and systemic and endocrine causes had been clinically evaluated and excluded during routine work-up, including basic laboratory testing such as complete blood count, biochemical parameters, thyroid function tests, and vitamin levels. These patients were clinically followed over time, with no signs or symptoms of increased intracranial pressure developing during follow-up. Evaluations in control patients were performed as part of routine clinical practice based on clinical indications; therefore, the standardized diagnostic protocol applied to idiopathic intracranial hypertension patients was not uniformly used in this group.

In total, 62 children with pediatric idiopathic intracranial hypertension and 62 controls (*n*=124) were included in the study. Sex distribution was comparable between groups, whereas age was not individually matched.

### Imaging parameters

All pediatric brain MRIs were performed on a Philips 3-T imaging system with a dedicated head coil (Philips Medical Systems, Best, the Netherlands). All studies included 3-dimensional (D) magnetization-prepared rapid gradient-echo (MP-RAGE) (echo time (TE), 11–12; repetition time (TR), 680) with 1 mm (mm) slice thickness (ST), fast spin-echo (FSE) T2-weighted images (TE, 100–120; TR, 3,000–4,000) in axial, coronal, and sagittal planes with 3 mm ST, fluid-attenuated inversion recovery (FLAIR) (TE, 110; TR, 7,000–9,000; inversion time (TI), 2,000) in axial plane with 3 mm ST, diffusion-weighted imaging (TE, 70–110; TR, 7,000–9,000; *b* value, 0 and 1,000) with 3 mm ST. Also, a non-contrast time-of-flight (TOF) sequence for MRV was used to evaluate the dural sinus. MRV was performed using a non-contrast TOF sequence in all subjects, and no contrast-enhanced MRI or contrast-enhanced MRV sequences were included in the imaging protocol used for this study.

### Image analysis and quantitative measurements

All MR images belonging to the patient and control groups were retrieved from the hospital’s local Picture Archiving and Communication System (PACS) and exported for analysis. The exported images were evaluated using Radiant Digital Imaging and Communications in Medicine (DICOM) Viewer (Medixant, Poznan, Poland). Measurements were performed by radiologists with 5 years and 15 years of experience in neuroradiology (M.I.I., F.C.). The radiologists were blinded to group assignment. Inter-rater reliability was assessed separately for continuous and categorical variables. For continuous quantitative measurements, the intraclass correlation coefficient (ICC) was calculated and showed excellent agreement (ICC=0.91). For categorical variables (binary/ordinal MRI signs), Cohen’s kappa was used (weighted kappa for ordinal features where applicable), with excellent agreement overall (*κ*=0.93; 95% CI, 0.88–0.98). Any disagreements between the two readers were resolved by consensus review, and the consensus label/measurement was used for analysis. The following parameters were recorded: (i) optic nerve sheath diameter, (ii) pituitary gland height, (iii) posterior globe flattening, (iv) optic nerve tortuosity, (v) optic nerve protrusion, (vi) dural venous sinus abnormalities (none/hypoplasia/aplasia), (vii) transverse sinus stenosis, (viii) presence of arachnoid granulations, (ix) presence of slit ventricles, (x) Meckel’s cave diameter, (xi) cervical subcutaneous fat thickness (at the level of the C2 vertebral body), (xii) foramen magnum diameter, and (xiii) presence of inferior tonsillar displacement. All quantitative parameters were measured in millimeters, while qualitative parameters were recorded as “present/absent.” When necessary, reconstruction algorithms were utilized to optimize image planes for accurate assessment.

The measurements were performed with guidance from similar studies in the literature [14, 15, 18, 20]. Cervical fat tissue thickness, a new morphological parameter used in adults, was also applied to the pediatric population in our study [15]. The transverse and vertical diameters of the optic nerve sheath complex were measured in the coronal plane on the first slice following the ocular globe in T2-weighted images. Pituitary gland height was measured on the mid-sagittal plane on T1-weighted images; optic nerve tortuosity and protrusion were evaluated on axial and sagittal planes; and slit ventricles and Meckel’s cave diameter were measured on axial T2-weighted images. Mid-sagittal T1-weighted volumetric images were used to evaluate cervical subcutaneous fat thickness, transverse and anteroposterior diameters of the foramen magnum, and the presence of inferior tonsillar displacement. MRV images were utilized to assess dural venous sinus abnormalities, transverse sinus stenosis, and the presence of arachnoid granulations.

The maximum axial diameter of Meckel’s cave was measured. Cervical subcutaneous fat thickness was assessed at the level of the C2 vertebral body by measuring the posterior subcutaneous fat tissue. Complete empty sella was defined as >50% of the sella filled with CSF or a pituitary gland height ≤2 mm, whereas partial empty sella was defined as <50% CSF filling with a pituitary gland height ≥3 mm; for pituitary heights between 2 mm and 3 mm, classification was determined by the CSF-filling criterion (<50% as partial and >50% as complete) [21]. When necessary, volumetric sequences were reconstructed to obtain planes parallel to the foramen magnum for accurate measurement of its diameters. For inferior tonsillar displacement, any measurable tonsillar descent was recorded as present/absent (binary). Tonsillar herniation was defined as >5 mm descent; no patient met the herniation threshold [22].

### Exploratory data analysis

Table 1 presents the distribution of all demographic and MRI-derived quantitative variables obtained in the study between the control group (class 0) and the pediatric idiopathic intracranial hypertension group (class 1), along with the statistical results comparing the two groups. Welch’s *t*-test, the Mann–Whitney *U* test, Levene’s variance test, and standardized effect size measures (Cohen’s *d* and Cliff’s delta) were used in these comparisons. For each variable, we used Welch’s *t*-test when Levene’s test did not indicate significant variance heterogeneity (*P*≥0.05) and the Mann–Whitney *U* test otherwise. The resulting *P*-value was designated as the primary *P*-value and subjected to multiple-comparison correction using the Benjamini–Hochberg FDR and Holm–Bonferroni procedures. Dural venous sinus abnormality was encoded as a three-level ordinal variable (1=none, 2=hypoplasia, 3=aplasia) capturing any structural abnormality of the dural venous sinuses. In addition, transverse venous sinus stenosis was modelled as a separate binary variable (1=No, 2=Yes) to specifically indicate the presence of focal transverse sinus narrowing.

**Table 1:** Group-wise distribution and statistical comparison of demographic and magnetic resonance imaging-derived features between control participants (class 0) and pediatric idiopathic intracranial hypertension patients (class 1)

Feature	n class0	n class1	Mean class0	Mean class1	Median class0	Median class1	Delta mean (1-0)	Welch *P* (two-sided)	Mann–Whitney *P* (two-sided)	Levene *P*	Cohen’s *d*	Cliff’s delta	Primary *P*	FDR-adjusted *P* (BH)	Holm-adjusted *P*	Sig. (FDR-adjusted *P*<0.01)
Age (years)	62	62	12.58	11.4	14	11	−1.18	0.07	0.07	0.62	−0.33	−0.09	0.07	0.1	0.7	0
Sex (1=male, 2=female)	62	62	1.6	1.69	2	2	0.1	0.26	0.26	0.26	0.2	0.02	0.26	0.28	1	0
Body weight (kg)	62	62	48.82	53.15	48.5	53	4.33	0.23	0.32	0.21	0.22	0.21	0.32	0.32	1	0
Height (cm)	62	62	152.7	147.7	156	151.5	−5	0.09	0.08	0.71	−0.3	0.14	0.08	0.11	0.73	0
Body mass index (BMI)	62	62	20.22	23.38	19.5	22.5	3.16	<0.01	<0.01	<0.01	0.56	0.25	<0.01	0.02	0.15	0
Right optic nerve sheath diameter (transverse, mm)	62	62	6.95	7.34	6.82	7.35	0.39	0.02	0.03	0.04	0.42	0.25	0.03	0.07	0.58	0
Right optic nerve sheath diameter (vertical, mm)	62	62	5.93	6.64	5.9	6.64	0.71	<0.01	<0.01	0.17	0.86	0.51	<0.01	<0.01	<0.01	1
Left optic nerve sheath diameter (transverse, mm)	62	62	6.87	7.3	6.83	7.27	0.43	0.01	0.02	0.09	0.45	0.5	0.02	0.06	0.44	0
Left optic nerve sheath diameter (vertical, mm)	62	62	5.97	6.49	5.96	6.52	0.51	<0.01	<0.01	0.28	0.59	0.49	<0.01	0.02	0.1	0
Pituitary gland thickness (mm)	62	62	5.52	4.3	5.54	4.32	−1.23	<0.01	<0.01	0.11	−0.91	−0.66	<0.01	<0.01	<0.01	1
Empty sella (1=none, 2=partial, 3=complete)	62	62	1.08	1.61	1	1	0.53	<0.01	<0.01	<0.01	0.89	0.33	<0.01	<0.01	<0.01	1
Posterior globe flattening (1=no, 2=yes)	62	62	1	1.35	1	1	0.35	<0.01	<0.01	<0.01	1.04	0.35	<0.01	<0.01	<0.01	1
Optic nerve tortuosity (1=no, 2=yes)	62	62	1.18	1.4	1	1	0.23	<0.01	<0.01	<0.01	0.51	0.34	<0.01	0.02	0.13	0
Optic nerve protrusion (1=no, 2=yes)	62	62	1	1.06	1	1	0.06	0.04	0.04	0.04	0.37	0.06	0.04	0.08	0.66	0
Dural venous sinus abnormality	62	62	1.55	1.74	1	2	0.19	0.2	0.09	0.2	0.23	0.47	0.09	0.12	0.75	0
Transverse venous sinus stenosis (1=no, 2=yes)	62	62	1.08	1.35	1	1	0.27	<0.01	<0.01	<0.01	0.7	0.31	<0.01	<0.01	<0.01	1
Arachnoid granulation (1=no, 2=yes)	62	62	1.1	1.16	1	1	0.06	0.29	0.29	0.29	0.19	0.11	0.29	0.3	1	0
Slit ventricle (1=no, 2=yes)	62	62	1.02	1.11	1	1	0.1	0.03	0.03	0.03	0.4	0.11	0.03	0.07	0.56	0
Right Meckel’s cave diameter (mm)	62	62	4.13	4.65	4.08	4.38	0.52	0.01	0.05	0.1	0.46	0.32	0.05	0.08	0.66	0
Left Meckel’s cave diameter (mm)	62	62	4.11	4.46	4.24	4.39	0.35	0.09	0.17	0.29	0.3	0.31	0.17	0.2	1	0
Neck fat tissue thickness (mm)	62	62	6.42	7.68	6.12	6.96	1.25	0.03	0.1	0.02	0.4	0.36	0.1	0.12	0.75	0
Foramen magnum diameter (transverse, mm)	62	62	22.87	23.89	22.75	24.05	1.02	0.03	0.06	0.1	0.39	0.39	0.06	0.1	0.7	0
Foramen magnum diameter (anteroposterior, mm)	62	62	16.74	16.16	16.3	15.8	−0.58	0.13	0.21	0.27	−0.27	−0.27	0.21	0.23	1	0
Inferior tonsillar displacement (1=no, 2=yes)	62	62	1.05	1.16	1	1	0.11	0.04	0.04	0.04	0.37	0.16	0.04	0.08	0.66	0

Class 0=control group (headache controls without papilledema and without structural intracranial pathology on MRI), Class 1=idiopathic intracranial hypertension patient group, Feature=evaluated MRI-derived variable, n class0 / n class1=number of valid observations in each group, Mean class0 / mean class1=group mean values, Median class0 / median class1=group median values, Delta means (1–0)=difference in group means (Class 1 − Class 0), Welch *P* (two-sided)= Welch’s t-test *P* value, Mann–Whitney *P* (two-sided)= Mann–Whitney U test *P* value, Levene *P* = Levene’s test *P* value for equality of variances, Cohen’s d=standardized effect size, Cliff’s delta=non-parametric effect size, Primary *P* = *P* value selected for multiple-comparison correction, *FDR*= false discovery rate, FDR-adjusted* P *(*BH*)=Benjamini–Hochberg false discovery rate-adjusted *P* value, Holm-adjusted *P*=Holm–Bonferroni corrected *P* value, Sig. (FDR-adjusted *P*<0.01)=significance indicator after false discovery rate correction, *MRI* magnetic resonance imaging

**Fig. 1:**
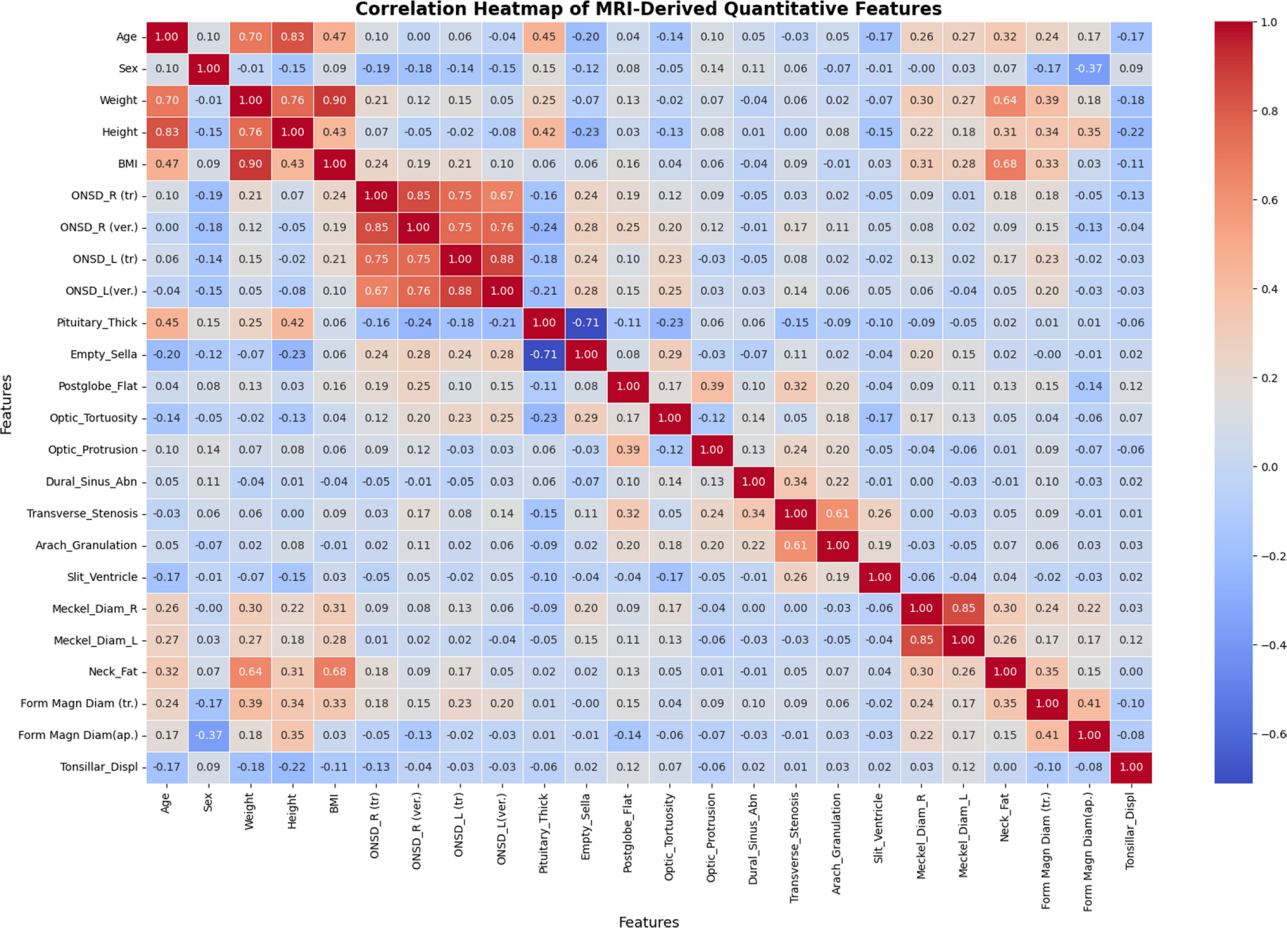
Correlation heatmap of all magnetic resonance imaging-derived features showing pairwise correlations across the full cohort. *BMI* body mass index, *ONSD_R (tr.)* right optic nerve sheath diameter (transverse, mm), *ONSD_R (ver.)* right optic nerve sheath diameter (vertical, mm), *ONSD_L (tr.)* left optic nerve sheath diameter (transverse, mm), *ONSD_L (ver.)* left optic nerve sheath diameter (vertical, mm), *Pituitary_Thick* pituitary gland thickness (mm), *Empty_Sella* empty sella, *Postglobe_Flat* posterior globe flattening,* Optic_Tortuosity* optic nerve tortuosity, *Optic_Protrusion* optic nerve protrusion, *Dural_Sinus_Abn* dural venous sinus abnormality, *Transverse_Stenosis* transverse venous sinus stenosis, *Arach_Granulation* arachnoid granulation,* Slit_Ventricle* slit ventricle, *Meckel_Diam_R* right Meckel’s cave diameter (mm), *Meckel_Diam_L* left Meckel’s cave diameter (mm), *Neck_Fat* neck fat tissue thickness (mm), *Form Magn Diam (tr.)* foramen magnum diameter (transverse, mm), *Form Magn Diam (ap.)* foramen magnum diameter (anteroposterior, mm), *Tonsillar_Displ inferior* tonsillar displacement

The results demonstrate pronounced differences in structural markers known to be associated with idiopathic intracranial hypertension, particularly optic nerve sheath measurements, pituitary gland thickness, empty sella findings, and transverse venous sinus stenosis. Together, these findings support the discriminative potential of MRI-derived metrics in this patient population. In addition, the correlation heatmap presented in Fig. 1 reveals clear collinearity clusters among optic nerve sheath measurements and Meckel’s cave diameters, suggesting that pediatric idiopathic intracranial hypertension is associated with coordinated structural alterations across related anatomical compartments. Supplementary Material 1 provides group-wise boxplots that visually illustrate the distributional differences of these quantitative MRI parameters between the control and idiopathic intracranial hypertension groups, highlighting that variables such as optic nerve sheath dimensions, empty sella degree, and posterior globe flattening exhibit markedly higher values in the patient group.

## Methods

We developed a supervised machine-learning pipeline to distinguish pediatric idiopathic intracranial hypertension cases from control patients. Data were arranged in a tabular format, encoding the class label (0=control, 1=pediatric idiopathic intracranial hypertension). All 24 demographic and MRI-derived features listed in Table 1 were initially included as candidate predictors. The binary group indicator (“Group,” 0=control, 1=pediatric idiopathic intracranial hypertension) served as the target variable and was not used as a predictor. For all features, complete data were available in both groups (*n*=62 per class); therefore, no missing-value imputation was required. Model performance was assessed using a repeated nested design with 20 outer repetitions. In each repetition, a stratified split assigned 20% of the data to an independent test set and 80% to the training and validation set. The training and validation set was further split into an inner training set (65% of the full cohort) and an inner validation set (15%), and all model selection and tuning were restricted to these inner subsets; the outer test set remained unseen until final evaluation.

All features were preprocessed using robust scaling, where median centering and interquartile range scaling were computed solely from the inner training set to strictly avoid data leakage. Within each outer repetition, we trained six classifier families: random forest [23], support vector machine (SVM) [24], multilayer perceptron (MLP) [25], extreme gradient boosting (XGBoost) [26], *k*-nearest neighbors (KNN) [27], and a Bagging ensemble [28]. For each model, Bayesian hyperparameter optimization with Optuna [29] (150 trials per model per repetition) was used to jointly tune model-specific hyperparameters and the number of selected features between 3 and 15, within a unified pipeline comprising robust scaling, univariate ANOVA *F*-test feature selection, and the classifier. The optimization objective was the F1-score for the pediatric idiopathic intracranial hypertension class on the inner validation set. The common Optuna workflow settings and the complete model-specific hyperparameter search spaces are provided in Supplementary Material 2.

For each model and repetition, the best-performing configuration was then retrained as a final pipeline on the combined inner training and validation data and evaluated once on the corresponding outer test set. From test predictions and predicted probabilities for idiopathic intracranial hypertension, we computed confusion matrices and derived the F1-score, the area under the receiver operating characteristic curve (AUC), average precision, sensitivity, specificity, precision, accuracy, Matthews correlation coefficient, and geometric mean of sensitivity and specificity. Test metrics were aggregated across the 20 repetitions to obtain means, standard deviations, and 95% confidence intervals. Between-model comparisons were performed using Friedman tests followed, when indicated, by Wilcoxon signed-rank tests with Holm correction; for AUC, paired *t*-tests with Holm adjustment were additionally applied. Finally, for a subset of representative runs (with test average precision close to the model-specific median), we applied Shapley additive explanations (SHAP) [30] and local interpretable model-agnostic explanations (LIME) [31] to the trained pipelines to qualitatively identify MRI-derived features that most strongly contributed to discrimination between pediatric idiopathic intracranial hypertension and controls.

An overview of the machine-learning pipeline, including the repeated nested design, Optuna-based Bayesian optimization, feature selection, model training, and evaluation workflow, is provided in Supplementary Material 3. All source code required to reproduce the full pipeline, including data preprocessing, hyperparameter optimization, model training, evaluation, and explainability analyses, is publicly available upon acceptance. All analyses and model training were implemented in Python.

## Results

Supplementary Material 4 displays the distribution of effect sizes based on Cohen’s *d* and Cliff’s *δ*, showing that right optic nerve sheath short axis, right optic nerve sheath diameter (coronal), transverse venous sinus stenosis, and empty sella exhibit the strongest discriminative effects between the pediatric idiopathic intracranial hypertension and control groups. These variables demonstrate consistently high effect sizes across parametric and non-parametric measures, indicating meaningful anatomical differences associated with elevated intracranial pressure. In contrast, features such as age, height, and foramen magnum diameter show minimal effect sizes, suggesting that they do not meaningfully differentiate between patients and controls.

Supplementary Material 5 presents the volcano plot highlighting variables that remain statistically significant after FDR correction. After FDR correction, posterior globe flattening, empty sella, right optic nerve sheath short axis, and transverse venous sinus stenosis clearly emerged with both high statistical significance and substantial effect sizes. Right optic nerve sheath diameter in the coronal plane showed a trend toward significance with a moderate effect size. Together, these findings reinforce their potential role as robust imaging markers of pediatric idiopathic intracranial hypertension. In contrast, most other MRI-derived features cluster below the FDR<0.01 threshold and exhibit modest Cohen’s *d* values, indicating limited group-separating power.

The overall performance of the machine-learning models is summarized in Table 2. The reported F1-scores and PR-AUC values throughout the “Results” section are computed with pediatric idiopathic intracranial hypertension as the positive class. All six classifiers achieved broadly similar performance in distinguishing pediatric idiopathic intracranial hypertension from controls. Mean accuracy ranged from approximately 70% to 74%, with KNN and MLP reaching 73.8%, Bagging 73.4%, and random forest and XGBoost 71.2% and 70.6%, respectively. For F1-score, MLP (72.9±11.0%) and SVM (72.5±10.8%) achieved the highest mean values, whereas Bagging, KNN, XGBoost, and random forest clustered in the 69–71% range with largely overlapping 95% confidence intervals. G-mean and MCC indicated moderate discriminative ability across models. MLP, Bagging, KNN, and XGBoost all achieved comparable trade-offs between sensitivity and specificity, whereas SVM showed markedly higher variability across folds. Sensitivity values were generally lower than specificity (recall ~63–72% vs. specificity ~69–84%), indicating that all models tended to protect controls slightly better than they detected pediatric idiopathic intracranial hypertension cases. In terms of PR-AUC and AUC, Bagging, random forest, XGBoost, and MLP all reached mean PR-AUC values around 83–84% with overlapping confidence intervals, while KNN trailed only slightly behind and SVM showed clearly lower and more variable performance. Run‑by‑run trajectories of accuracy, F1‑score, G‑mean, MCC, precision, recall, and specificity across the 20 repetitions (Supplementary Material 6) were consistent with these summaries, showing that Bagging, KNN, MLP, and random forest fluctuated within a relatively narrow band around their mean performance, whereas XGBoost and especially SVM exhibited greater fold‑to‑fold variability with occasional marked drops, in line with their larger standard deviations.

**Table 2:** Summary of model performance across 20 repetitions

Mean±SD [95% CI], %	Accuracy (%)	F1 (%)	G-mean (%)	MCC (%)	Precision (%)	Recall (%)	Specificity (%)	PR-AUC (%)	AUC (%)
Bagging	73.40±7.71 [69.79–77.01.79.01]	70.72±9.39 [66.33–75.12.33.12]	72.44±8.40 [68.51–76.37.51.37]	48.11±15.13 [41.03–55.20.03.20]	78.37±9.37 [73.98–82.75.98.75]	66.06±13.59 [59.70–72.42.70.42]	80.83±10.44 [75.95–85.72.95.72]	84.05±8.79 [79.94–88.17.94.17]	81.49±9.69 [76.96–86.03.96.03]
KNN	73.80±11.35 [68.49–79.11.49.11]	70.07±13.38 [63.81–76.33.81.33]	72.21±12.05 [66.57–77.85.57.85]	49.61±22.63 [39.01–60.20.01.20]	81.18±13.83 [74.71–87.66.71.66]	63.53±16.99 [55.57–71.48.57.48]	84.07±14.01 [77.51–90.63.51.63]	80.88±10.49 [75.97–85.79.97.79]	79.58±9.75 [75.02–84.15.02.15]
MLP	73.80±11.20 [68.56–79.04.56.04]	72.93±11.01 [67.77–78.08.77.08]	73.29±11.33 [67.99–78.59.99.59]	48.35±22.78 [37.68–59.01.68.01]	76.37±12.98 [70.30–82.44.30.44]	70.83±12.94 [64.78–76.89.78.89]	76.79±15.10 [69.73–83.86.73.86]	83.18±7.49 [79.67–86.69.67.69]	81.55±9.31 [77.20–85.91.20.91]
Random forest	71.20±9.59 [66.71–75.69.71.69]	69.11±8.91 [64.94–73.28.94.28]	70.35±9.36 [65.97–74.73.97.73]	43.39±19.81 [34.12–52.66.12.66]	76.42±12.88 [70.39–82.45.39.45]	64.04±9.70 [59.50–68.58.50.58]	78.14±14.57 [71.32–84.96.32.96]	83.41±9.85 [78.80–88.02.80.02]	80.75±10.36 [75.91–85.60.91.60]
SVM	70.40±14.65 [63.54–77.26.54.26]	72.51±10.81 [67.13–77.88.13.88]	59.71±32.02 [44.72–74.68.72.68]	53.95±20.76 [42.88–65.01.88.01]	79.50±18.01 [70.54–88.46.54.46]	63.85±26.96 [51.23–76.46.23.46]	77.63±30.57 [63.32–91.94.32.94]	73.31±20.77 [63.59–83.03.59.03]	66.83±26.39 [54.48–79.18.48.18]
XGBoost	70.60±9.65 [66.08–75.12.08.12]	70.26±9.94 [65.61–74.91.61.91]	66.24±18.08 [57.78–74.70.78.70]	45.91±17.70 [37.38–54.45.38.45]	74.21±14.69 [67.34–81.09.34.09]	71.67±19.11 [62.72–80.61.72.61]	69.42±24.41 [58.00–80.85.00.85]	83.26±6.59 [80.17–86.35.17.35]	80.53±7.95 [76.81–84.25.81.25]

*AUC* area under the receiver operating characteristic curve, *CI* confidence interval, *MCC* Matthews correlation coefficient, *PR-AUC* area under the precision–recall curve, *SD* standard deviation

**Fig. 2:**
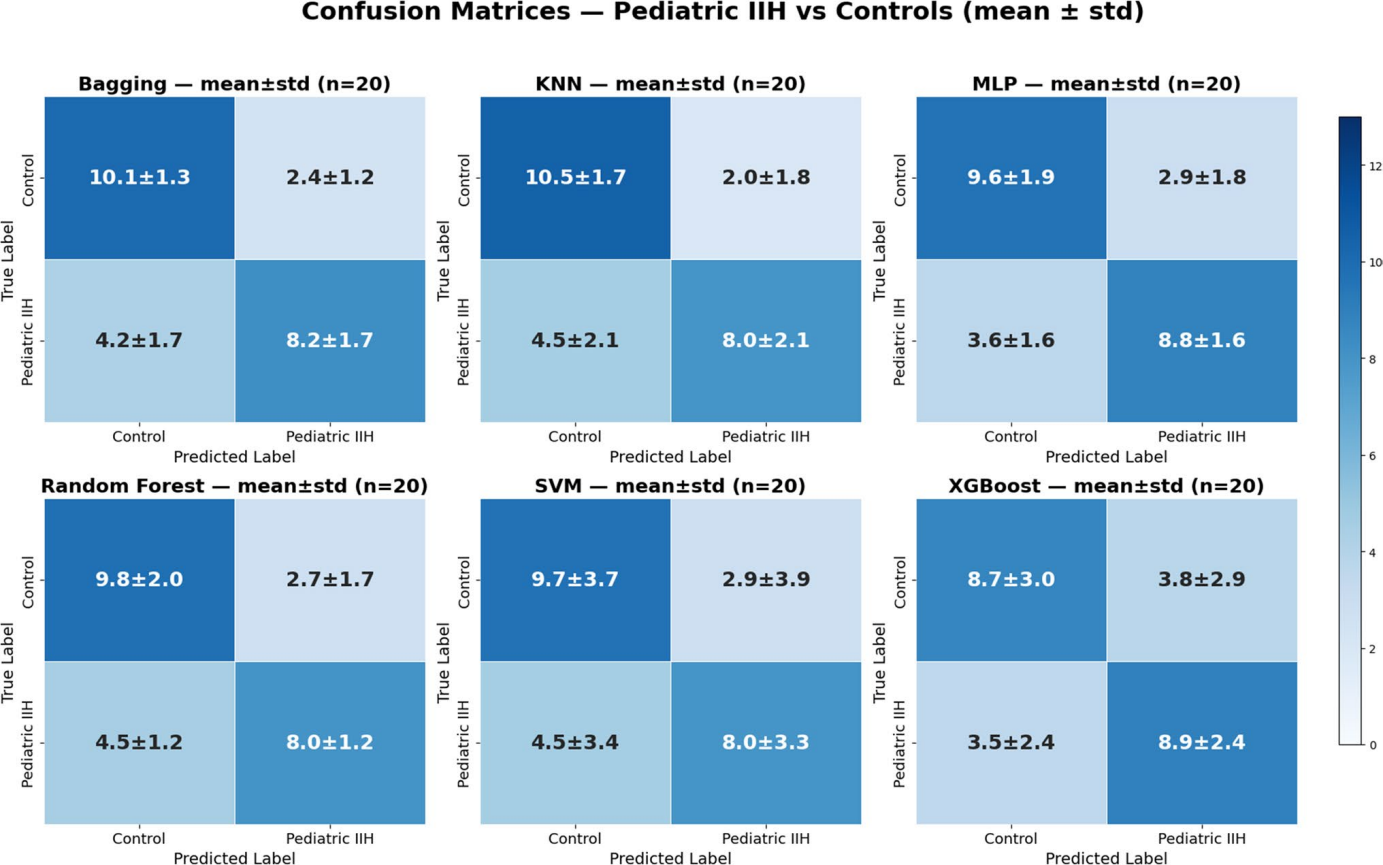
Mean±standard deviation confusion matrices across 20 independent repetitions for all models. *IIH* idiopathic intracranial hypertension, *KNN* *k*-nearest neighbors, *MLP* multilayer perceptron, *SVM* support vector machine, *XGBoost* extreme gradient boosting

The confusion matrices averaged over the 20 repetitions (Fig. 2) are consistent with these curve-level findings. In a typical test split, the classifiers correctly identified roughly 9–11 control subjects and 8–9 pediatric idiopathic intracranial hypertension cases, while misclassifying about 2–3 controls as idiopathic intracranial hypertension (false positives) and 3–5 idiopathic intracranial hypertension cases as controls (false negatives). Thus, differences between models are quantitative rather than qualitative; some models (e.g., MLP and XGBoost) tend to reduce false negatives at the cost of slightly more false positives, whereas Bagging and KNN show marginally higher specificity with a small increase in missed idiopathic intracranial hypertension cases.

**Fig. 3:**
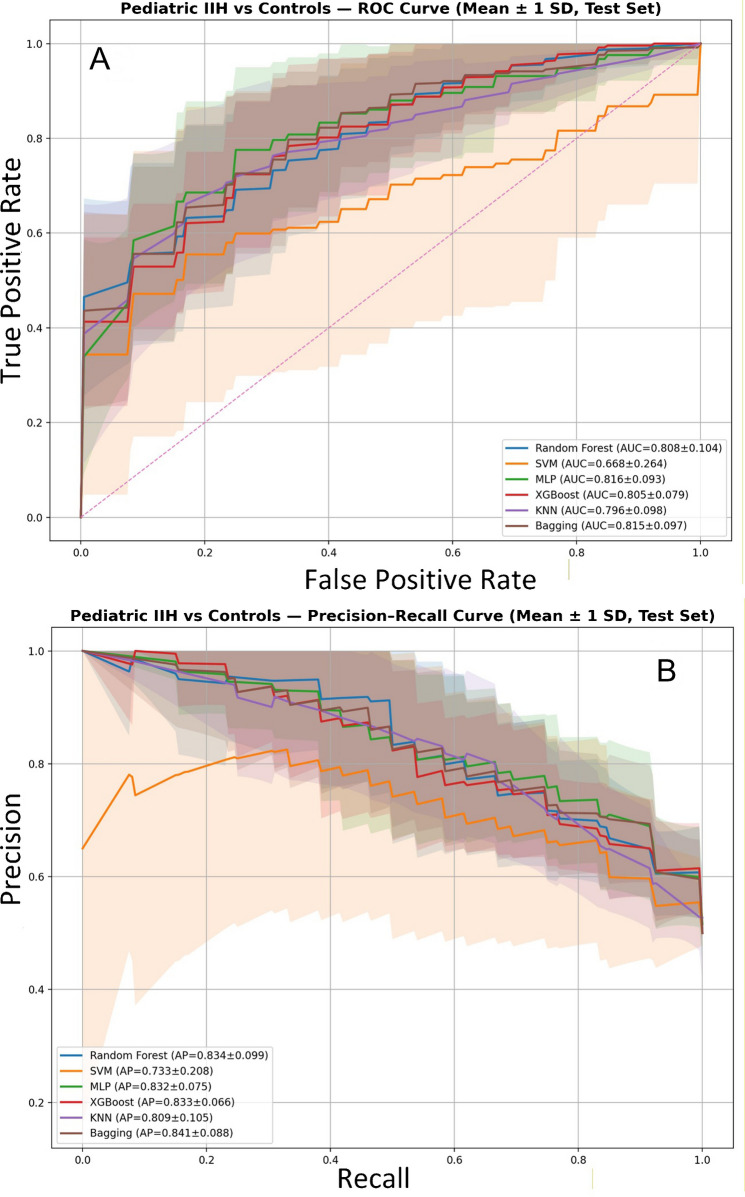
Receiver operating characteristic and precision–recall curves for pediatric idiopathic intracranial hypertension versus controls across 20 independent test repetitions. **a** Mean receiver operating characteristic curves with ±1 standard deviation shading for all six classifiers. **b** Mean precision–recall curves with ±1 standard deviation shading. *ROC* receiver operating characteristic, *AUC* area under the receiver operating characteristic curve, *SD* standard deviation, *IIH* idiopathic intracranial hypertension, *KNN* *k*-nearest neighbors, *MLP* multilayer perceptron, *SVM* support vector machine, *XGBoost* extreme gradient boosting

The receiver operating characteristic (ROC) curves for Bagging, MLP, random forest, XGBoost, and KNN almost completely overlap, with true-positive rates around 0.8 at a false-positive rate of 0.2–0.3 and mean AUC values between 0.80 and 0.82 (Fig. 3a). SVM lies consistently below the other curves and shows a much wider shaded area, reflecting lower and more unstable discrimination (AUC 0.67±0.26). The mean ROC and precision–recall curves, averaged over the 20 test folds, are depicted in Fig. 3b. The precision–recall curves confirm that all non-SVM models maintain high precision (typically around 0.75–0.85) across a broad range of recall values up to about 0.5–0.6, followed by a gradual decline as recall approaches 1.0. Bagging, MLP, random forest, and XGBoost display very similar PR profiles with mean average precision values around 0.83–0.84, whereas SVM again shows both lower precision at a given recall and larger variability (average precision 0.73±0.21).

Pairwise statistical comparisons confirmed that none of these apparent trends reached statistical significance after correction for multiple testing. Wilcoxon signed-rank tests with Holm adjustment, summarized for F1-score in Table 3 and for the remaining metrics in Supplementary Material 7, revealed no significant pairwise differences between models for accuracy, F1-score, G-mean, MCC, precision, recall, or specificity (all Holm-adjusted *P*≥0.18). Likewise, paired *t*-tests on AUC did not identify any statistically significant superiority of one model over another after Holm correction (Supplementary Material 7). Taken together, Table 2, Figs. 2 and 3, and Table 3 indicate that, within the limits of the sample size and variability of repeated splits, Bagging, KNN, MLP, random forest, and XGBoost offer broadly comparable and clinically usable discrimination for pediatric idiopathic intracranial hypertension, whereas SVM shows more variable performance but is not significantly inferior in formal pairwise testing. Supplementary Material 6 further supports this interpretation by illustrating the fold‑wise stability of all evaluation metrics across the 20 repetitions.

**Table 3:** Pairwise Wilcoxon–Holm tests for F1-score across 20 repetitions (All pairwise comparisons showed no statistically significant differences after Holm correction (adjusted *P*≥0.05), indicating no model consistently outperformed others in terms of F1-score)

Metric	Comparison	Δ Mean points	Wilcoxon *P*	Holm-adjusted *P*	sig
F1-score	Bagging vs. KNN	1.84	0.52	1	ns
	Bagging vs. MLP	−3.01	0.25	1	ns
	Bagging vs. random forest	0.05	0.94	1	ns
	Bagging vs. SVM	−2.20	0.54	1	ns
	Bagging vs. XGBoost	−0.32	0.97	1	ns
	KNN vs. MLP	−4.85	0.20	1	ns
	KNN vs. random forest	−1.78	0.64	1	ns
	KNN vs. SVM	−4.03	0.17	1	ns
	KNN vs. XGBoost	−2.16	0.70	1	ns
	MLP vs. random forest	3.06	0.39	1	ns
	MLP vs. SVM	0.81	0.44	1	ns
	MLP vs. XGBoost	2.69	0.52	1	ns
	Random forest vs. SVM	−2.25	0.65	1	ns
	Random forest vs. XGBoost	−0.38	0.87	1	ns
	SVM vs. XGBoost	1.87	0.55	1	ns

*ns* not significant, *sig* significant

**Fig. 4:**
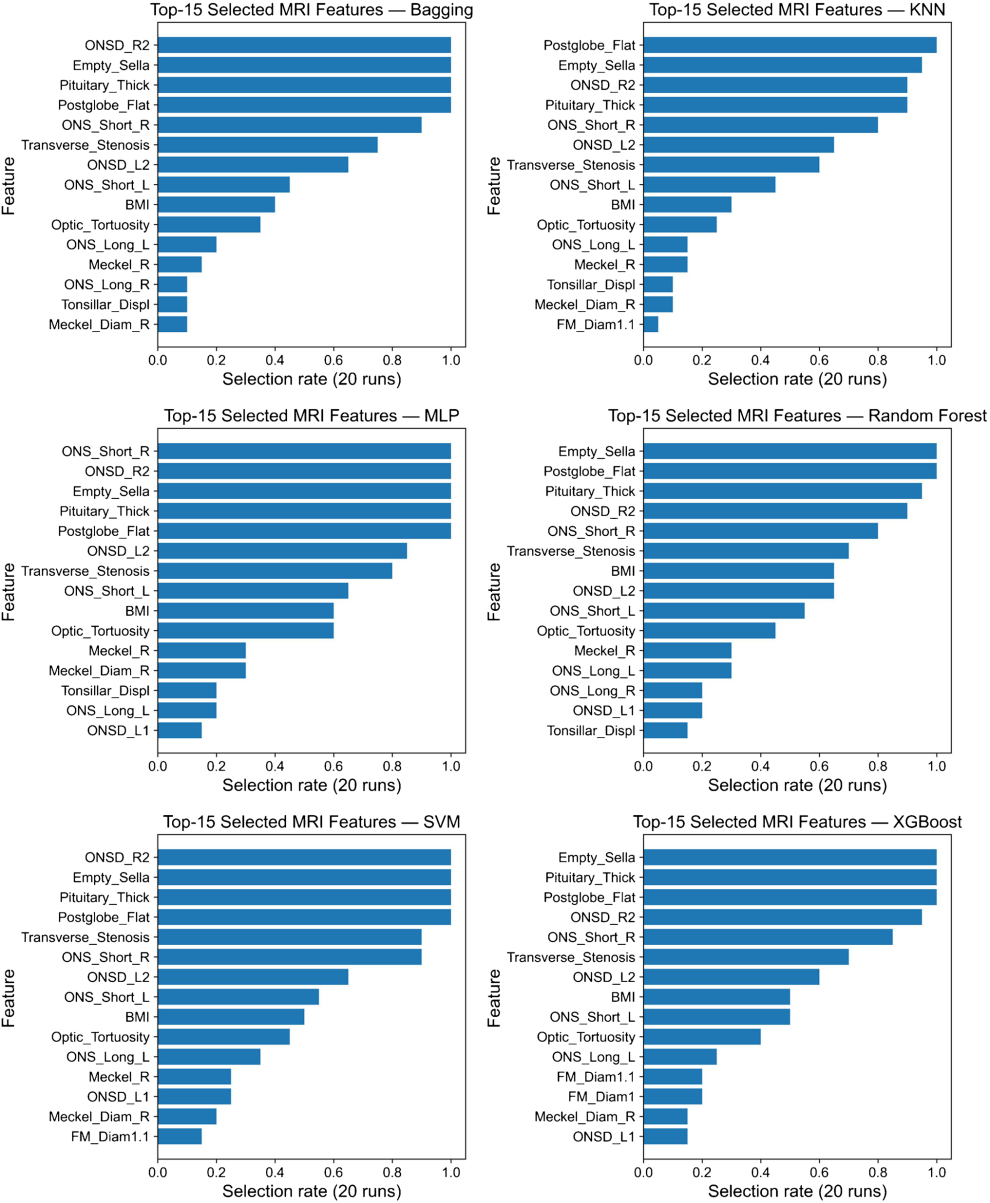
Top-15 most frequently selected magnetic resonance imaging-derived features across six machine-learning models, ranked by selection frequency across 20 repetitions for pediatric idiopathic intracranial hypertension classification. *KNN* *k*-nearest neighbors, *MLP* multilayer perceptron, *SVM* support vector machine, *XGBoost* extreme gradient boosting, *BMI* body mass index, *Empty_Sella* empty sella, *FM_Diam* Foramen magnum diameter, *Meckel_Diam* Meckel’s cave diameter (mm), *ONSD* optic nerve sheath diameter, *Optic_Tortuosity* optic nerve tortuosity, *Pituitary_Thick* pituitary gland thickness, *Postglobe_Flat* posterior globe flattening, *Tonsillar_Displ* inferior tonsillar displacement, *Transverse_Stenosis* transverse venous sinus stenosis, *IIH* idiopathic intracranial hypertension

Consistent with the univariate effect-size and FDR analyses described above (Supplementary Material 4), the model-based feature-selection frequencies showed a convergence across all six classifiers (Fig. 4). The variables that exhibited the largest effect sizes and remained significant after FDR correction, posterior globe flattening, empty sella, right optic nerve sheath diameter (coronal), right optic nerve sheath short axis, and transverse venous sinus stenosis were also among the most frequently selected features in the multivariate *k*-best procedure, typically appearing in more than 70–80% of runs for almost every model. Pituitary gland thickness and bilateral optic nerve sheath dimensions (e.g., left optic nerve sheath diameter, left optic nerve sheath short axis, left optic nerve sheath long axis) likewise showed high selection rates, further supporting their relevance as imaging correlates of elevated intracranial pressure. In contrast, variables that showed negligible effect sizes in the exploratory analysis, such as age, height, and foramen magnum diameters, were rarely chosen by the feature selector and almost never appeared in the top-15 lists for any classifier. This close agreement between Supplementary Material 4–5 and the selection-frequency plots in Fig. 4 indicates that the machine-learning pipeline consistently prioritized a core set of anatomically plausible MRI markers of pediatric idiopathic intracranial hypertension while down-weighting features with limited group-separating power.

**Fig. 5:**
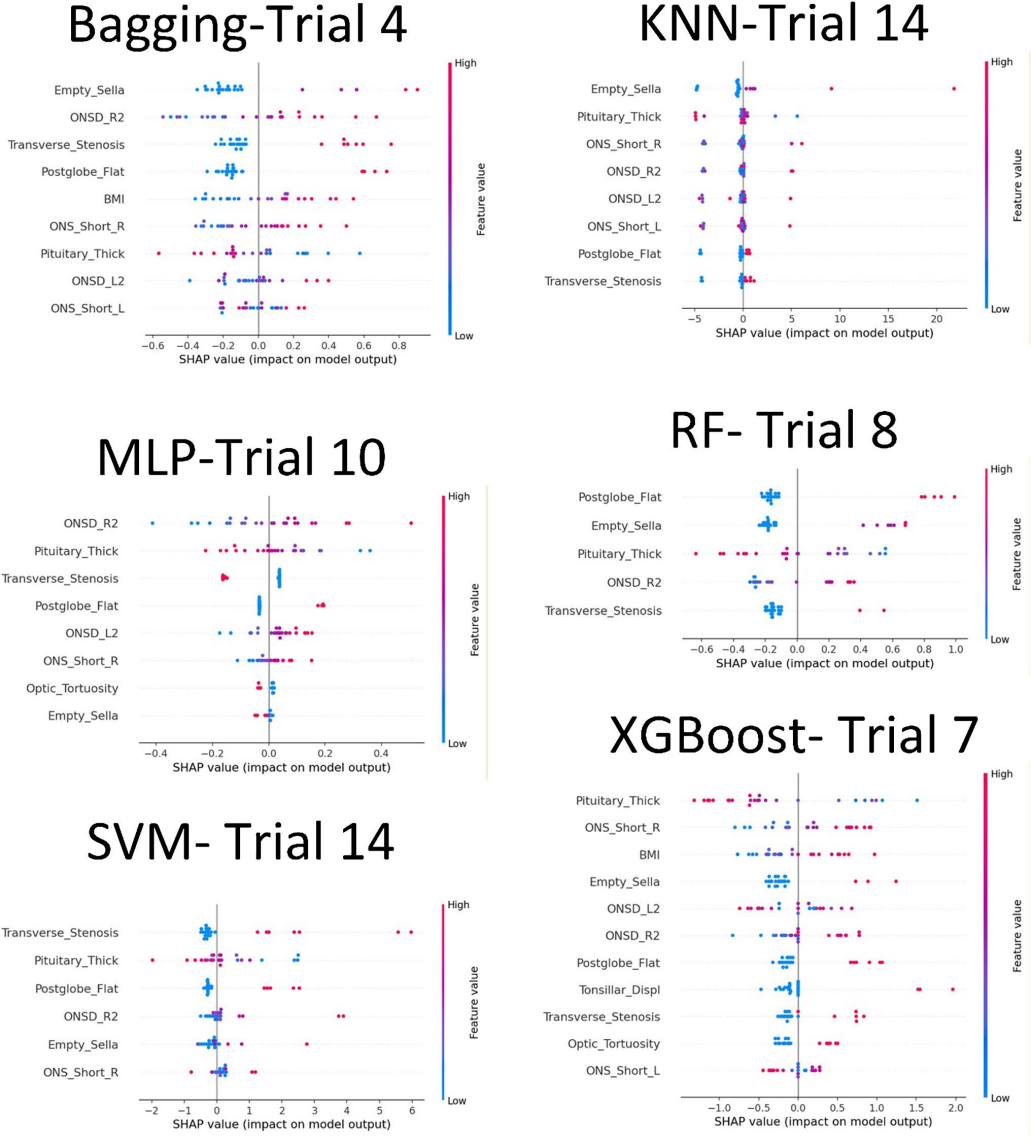
Shapley additive explanations (*SHAP*) summary plots (beeswarm) for representative runs of each model. Representative trials: Bagging (Trial 4), *k*-nearest neighbors (*KNN*; Trial 14), multilayer perceptron (*MLP*; Trial 10), random forest (*RF*; Trial 8), support vector machine (*SVM*; Trial 14), and extreme gradient boosting (*XGBoost*; Trial 7). *BMI* body mass index, *Empty_Sella* empty sella, *ONSD* optic nerve sheath diameter, *Optic_Tortuosity* optic nerve tortuosity, *Pituitary_Thick* pituitary gland thickness, *Postglobe_Flat* posterior globe flattening, *Tonsillar_Displ* inferior tonsillar displacement, *Transverse_Stenosis* transverse venous sinus stenosis

**Fig. 6:**
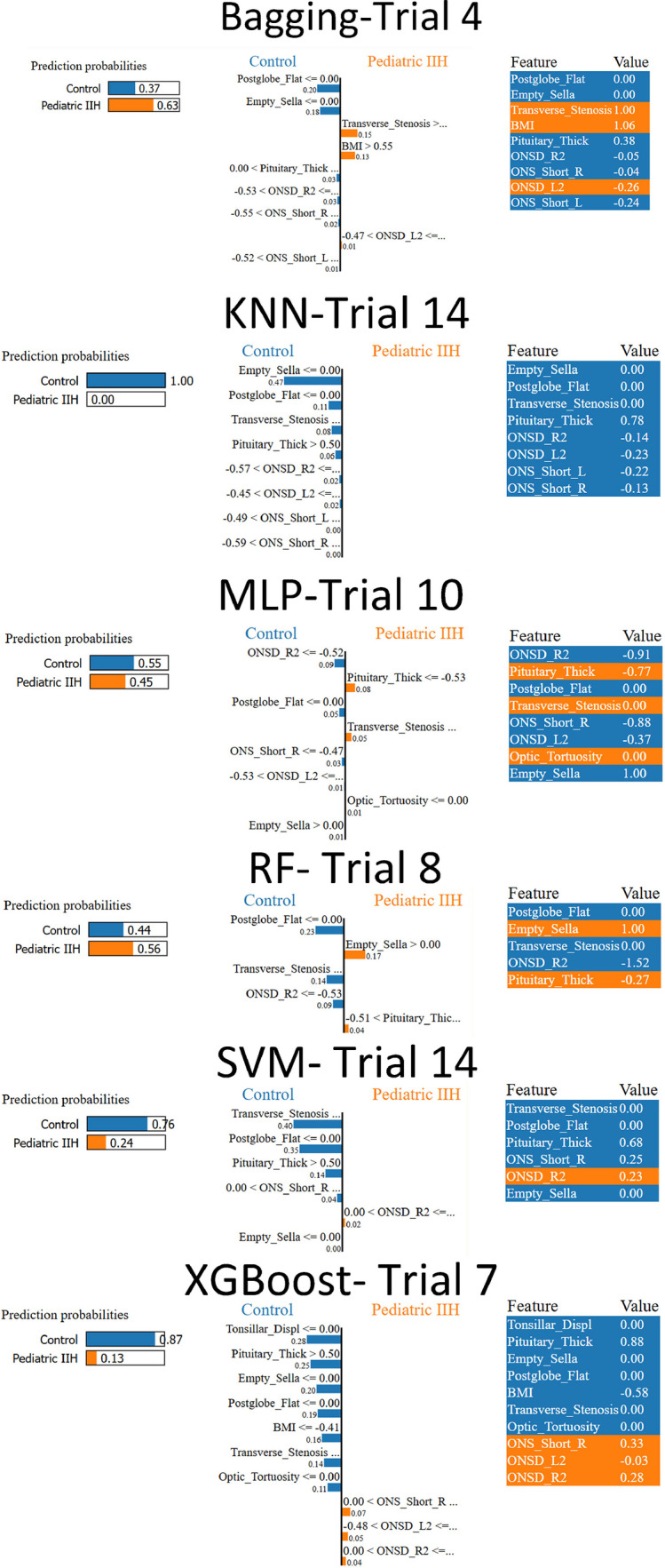
Local interpretable model-agnostic explanations (LIME) for the same representative test instances used in the Shapley additive explanations analysis. Representative trials: Bagging (Trial 4), *k*-nearest neighbors (*KNN*; Trial 14), multilayer perceptron (*MLP*; Trial 10), random forest (*RF*; Trial 8), support vector machine (*SVM*; Trial 14), and extreme gradient boosting (*XGBoost*; Trial 7). *BMI* body mass index, *Empty_Sella* empty sella, *ONSD* optic nerve sheath diameter, *Pituitary_Thick* pituitary gland thickness, *Postglobe_Flat* posterior globe flattening, *Transverse_Stenosis* transverse venous sinus stenosis, *Optic_Tortuosity* optic nerve tortuosity, *Tonsillar_Displ* inferior tonsillar displacement

To further characterize how individual MRI variables influenced the classifiers, we applied SHAP (Fig. 5) and LIME (Fig. 6) to representative high-performing trials of each model. Both methods converged on a compact set of drivers: Posterior globe flattening, empty sella, pituitary gland thickness, body mass index (BMI), optic nerve sheath measurements (right and left optic nerve sheath diameter in the coronal plane and their long and short axes), and transverse venous sinus stenosis, with inferior tonsillar displacement and optic nerve tortuosity contributing in some models. Across Bagging, KNN, MLP, random forest, SVM, and XGBoost, higher BMI and enlarged peri-bulbar optic nerve sheath diameters, together with the presence of posterior globe flattening, empty sella and transverse venous sinus stenosis, consistently shifted predictions toward the pediatric idiopathic intracranial hypertension class, whereas their absence and smaller sheath dimensions favored the control class. The close agreement between SHAP, LIME, univariate effect sizes and feature-selection frequencies supports both the robustness and the anatomical plausibility of the MRI markers identified for pediatric idiopathic intracranial hypertension.

To further characterize how the models exploited the MRI feature space, we examined the distribution of hyperparameters among the best-performing trials (Supplementary Material 8). For random forest, Optuna consistently favored large ensembles with approximately 800–1800 trees and relatively deep trees (typical max_depth ~40–60), combined with small leaves and splits (min_samples_leaf ~2–10, min_samples_split ~6–35) and information-based criteria (entropy/log-loss) rather than Gini. SVM best models were dominated by RBF kernels, with C values in the moderate-to-high range (roughly tens to a few hundred) and *γ* either set to “scale” or tuned in the 10⁻^3^–10⁻^1^ band, indicating flexible but not excessively sharp decision boundaries. Optimal MLP configurations were compact networks with one to three hidden layers and ~50–300 units, mostly using tanh or logistic activations, small L2 penalties and relatively small learning rates, suggesting mild regularization and avoidance of overly large architectures. KNN solutions typically used small neighborhoods (between 3–8), distance weighting, and Minkowski-type metrics with exponents close to the Euclidean norm (between 2–4), in line with the local structure of the data. For Bagging, KNN base learners were more common than decision trees, with 150–350 base estimators and both row and feature subsampling in the 0.5–0.75 range, providing additional variance reduction on top of the individual weak learners. XGBoost best models generally used 900–2100 boosting rounds with moderately deep trees (max depth between 8–17), small-to-moderate learning rates, and non-zero *γ* plus light L1/L2 regularization and positive class weighting factor close to the empirical class imbalance.

## Discussion

This study shows that quantitative MRI measurements, analyzed within a rigorously validated machine-learning pipeline, can distinguish pediatric idiopathic intracranial hypertension from controls with moderate to good accuracy. Across six classifier families and 20 repeated nested cross-validation splits, mean AUC values clustered around 0.80–0.82 for Bagging, KNN, MLP, random forest, and XGBoost, with similar F1-scores and precision–recall profiles and no statistically significant pairwise differences after multiple-comparison correction. Feature-selection frequencies and SHAP and LIME explanations converged on a compact, anatomically coherent set of predictors centered on optic nerve sheath expansion, pituitary gland flattening/empty sella, posterior globe flattening, and transverse venous sinus stenosis, with additional contributions from BMI and selected posterior fossa findings. Overall, these findings suggest that MRI abnormalities traditionally considered supportive of idiopathic intracranial hypertension contain genuine discriminative information when quantified and analyzed jointly.

The imaging pattern highlighted by our models mirrors and extends previous qualitative descriptions of MRI changes associated with idiopathic intracranial hypertension. Empty sella, optic nerve sheath distension, posterior globe flattening, and transverse sinus stenosis have all been reported as markers of raised intracranial pressure in adults and mixed-age cohorts, but their diagnostic utility in children has been less clearly quantified. Notably, the core imaging markers prioritized by our models correspond closely to the supportive neuroimaging signs emphasized in the 2013 revised Friedman criteria for pseudotumor cerebri syndrome, including idiopathic intracranial hypertension. This alignment supports the clinical plausibility of the learned patterns. The present machine-learning framework goes beyond a rule-based checklist by quantifying these markers, integrating them jointly with additional routinely obtainable variables, and generating uncalibrated model outputs with interpretable feature attributions. In our pediatric cohort, these variables showed large univariate effect sizes, high selection frequencies across models, and consistent positive SHAP and LIME contributions toward the idiopathic intracranial hypertension class. By contrast, demographic variables such as age and height, craniometric measures of the foramen magnum, and several descriptors of Meckel’s cave and venous sinuses contributed little to model output. This pattern suggests that a relatively small subset of MRI markers captures most of the discriminative signal in pediatric idiopathic intracranial hypertension and may improve diagnostic confidence in borderline cases.

From a modelling perspective, no single algorithm clearly outperformed the others. Bagging, KNN, MLP, random forest, and XGBoost produced highly overlapping ROC and precision–recall curves, and even SVM, despite lower mean AUC and greater variability, was not significantly inferior in formal pairwise testing. Supplementary Material 6 was consistent with these findings, showing relatively stable performance for Bagging, KNN, MLP, and random forest, with greater fold-to-fold variability for XGBoost and especially SVM. Rather than identifying a single “winning” algorithm, these results suggest that the MRI feature space contains a stable, learnable decision boundary that can be captured by several classifier families when appropriate regularization and model selection are applied. Clinically, this is reassuring because centers with different technical expertise or software libraries may still be able to implement comparable decision-support tools without being tied to a single proprietary architecture.

Another important observation is that specificity generally exceeded sensitivity, indicating that the models protected controls slightly better than they detected idiopathic intracranial hypertension. This likely reflects both the underlying feature distributions, for example, empty sella and increased optic nerve sheath diameter can occur incidentally in some controls and the use of F1 optimization with idiopathic intracranial hypertension as the positive class. In practice, such a profile may still be useful: in a diagnostic pathway where lumbar puncture and neuroophthalmologic assessment remain the reference standards, a tool that flags patients with a high model score while maintaining a relatively low false-positive rate could help prioritize urgent work-up and surveillance without overwhelming services. At the same time, the observed false-negative rate (typically 3–5 missed idiopathic intracranial hypertension cases per test fold) underscores that MRI-based models should complement, not replace, clinical judgment and CSF opening pressure measurements.

Interpretability is central to the translational value of this work. SHAP and LIME recapitulated known pathophysiological relationships: higher BMI, enlarged retrobulbar optic nerve sheaths, posterior globe flattening, and empty sella all pushed predictions toward the idiopathic intracranial hypertension class. The close alignment between interpretability analyses, univariate effect sizes, and feature-selection frequencies suggests that the models are not relying primarily on spurious correlations, but instead on anatomically plausible markers of chronically elevated intracranial pressure. This adds face validity and may facilitate clinical acceptance of machine-learning decision aids in pediatric neuroophthalmology.

To place our results in the context of pediatric imaging literature, prior pediatric case–control and validation studies have mainly reported the diagnostic performance of individual MRI markers or simple rule-based combinations. In a pediatric case–control cohort, perioptic subarachnoid space diameter ≥5.2 mm was identified as an independent predictor of idiopathic intracranial hypertension with 87% sensitivity and 67% specificity [20]. In a larger pediatric pseudotumor cerebri syndrome cohort evaluating the Friedman neuroimaging criteria, the presence of ≥3 of 4 key MRI signs (pituitary gland flattening, posterior scleral flattening, optic nerve sheath distension, and transverse venous sinus stenosis) achieved 62% sensitivity and 95% specificity, while transverse venous sinus stenosis alone reached 74% sensitivity and 100% specificity [32]. Our multifeature machine-learning models showed a comparable high-specificity/moderate-sensitivity profile (Table 2) while leveraging continuous quantitative measurements and providing transparent feature attributions alongside uncalibrated model outputs, which may be useful when individual MRI signs are borderline or variably present in children.

Direct comparison with other diagnostic tools or scoring systems remains challenging because, in the pediatric setting, there is no universally adopted and externally validated radiologist-based MRI scoring system or clinical prediction rule with directly comparable discrimination metrics in similar cohorts. Moreover, most available pediatric imaging studies focus on individual MRI signs or simple rule-based combinations rather than standardized multiparametric frameworks. Accordingly, our data do not show that the proposed machine-learning approach improves diagnostic accuracy over conventional MRI-based assessment or expert radiologist interpretation, because no direct within-cohort head-to-head comparison was performed. Instead, its potential added value lies in standardizing the joint interpretation of multiple routinely obtainable MRI markers, incorporating continuous quantitative measurements, and providing interpretable, uncalibrated model scores. Future prospective multicenter studies should compare this approach directly with expert radiologist assessment and predefined conventional MRI-sign combinations to determine whether it offers incremental diagnostic benefit.

Several limitations should be acknowledged. First, this was a single-center retrospective study conducted in a tertiary care setting, and the MRI acquisition and measurement protocols reflect the practice of one institution. External generalizability to other scanners, sequences, and case mixes therefore remains to be established. Second, although the cohort is relatively large for pediatric idiopathic intracranial hypertension, it remains modest from a machine-learning perspective given the dimensionality of the feature space and the use of six classifier families. Our nested repeated cross-validation framework, strict separation of training, validation, and test data, and conservative statistical comparisons were designed to reduce overfitting, but some optimism bias cannot be excluded. Formal calibration analysis was not performed; therefore, model outputs should be interpreted as uncalibrated predicted probabilities for decision support rather than fully calibrated absolute risk estimates. Age was not individually matched between groups, standardized pubertal staging was not available, and model performance was not evaluated separately across age-defined subgroups. Consequently, a biologically grounded prepubertal-versus-postpubertal subgroup analysis could not be performed, and whether performance differs across pediatric age strata remains unknown. In our cohort, age showed only a small between-group effect size and a minor influence on model output. Third, several MRI measurements involved manual or semi-manual assessment, such as optic nerve sheath diameters and pituitary thickness, and are therefore subject to intra- and inter-observer variability in routine practice. In addition, cervical subcutaneous fat thickness at the C2 level should be considered exploratory in children and requires pediatric normative data and external validation.

Finally, the control group consisted of children with headache and no structural intracranial pathology on MRI rather than asymptomatic community controls. Although this makes the classification task more clinically realistic, it may limit applicability to other diagnostic settings. Because lumbar puncture was not clinically indicated in controls, intracranial pressure was not formally assessed, and subclinical or borderline cases, including idiopathic intracranial hypertension without papilledema, cannot be entirely excluded. Accordingly, some controls may have represented occult or borderline idiopathic intracranial hypertension. All lumbar punctures in the pediatric idiopathic intracranial hypertension group were performed under benzodiazepine sedation, and although MRI preceded lumbar puncture and no treatment for idiopathic intracranial hypertension had been initiated beforehand, sedation may still have influenced measured opening pressure in borderline cases. If present, this would be expected to bias against the inclusion of some true borderline idiopathic intracranial hypertension cases rather than to inflate case assignment. Among controls, 14 of 62 (22.6%) had one supportive MRI sign, 4 of 62 (6.5%) had two, and none had three or more. In addition, a fully standardized screening protocol for medication-related, endocrine, and other systemic secondary causes was not uniformly applied to all control patients, because evaluations were performed according to clinical indications.

Despite these limitations, the study has several strengths. The cohort was well-phenotyped with rigorous application of modified Dandy criteria and lumbar puncture thresholds, and the balanced case–control design avoided extreme class imbalance. The pipeline incorporated robust scaling, nested hyperparameter optimization, and multiple complementary performance metrics. The simultaneous evaluation of six algorithmic families, together with SHAP and LIME interpretability analyses, provides a broad view of what current tabular machine-learning methods can extract from conventional MRI in pediatric idiopathic intracranial hypertension. The convergence of quantitative, model-based, and qualitative radiologic evidence on the same anatomical markers suggests that the findings are unlikely to be artifacts of a single analytic choice.

Future work should extend this framework in several directions. Multicenter prospective studies with harmonized imaging protocols are needed to validate the identified MRI markers and machine-learning models across different scanners, populations, and prevalence settings. Incorporating additional modalities, such as optical coherence tomography, visual field indices, or detailed anthropometric and endocrine data, may improve discriminative performance and support integrated risk scores. Rather than selecting a single “best” algorithm, committee-based or hybrid classifiers that aggregate predictions from several well-calibrated models could also be explored to improve robustness and support flexible rule-in versus rule-out screening.

## Conclusion

This proof-of-concept study demonstrates that machine-learning analysis of routinely obtainable MRI markers can distinguish pediatric idiopathic intracranial hypertension from headache controls with moderate discriminatory performance and can identify a compact, anatomically plausible set of discriminative imaging predictors. These findings support the feasibility and potential of moving beyond purely qualitative MRI interpretation toward quantitatively informed, patient-specific uncalibrated predicted probabilities for decision support in pediatric practice. However, further prospective multicenter validation, refinement, and assessment of clinical workflow impact are required before such models can be considered for routine clinical implementation.

## Supplementary Information

Below is the link to the electronic supplementary material.

ESM 1 (DOCX 228 KB)

ESM 2 (DOCX 22.8 KB)

ESM 3 (DOCX 17.4 KB)

ESM 4 (DOCX 116 KB)

ESM 5 (DOCX 111 KB)

ESM 6 (DOCX 0.99 MB)

ESM 7 (DOCX 29.4 KB)

ESM 8 (DOCX 22.5 KB)

## Data Availability

The datasets generated and/or analysed during the current study are not publicly available. De-identified data may be made available by the corresponding author upon reasonable request.
