# Supplementary material for: Diagnostic value of machine-learning using conventional magnetic resonance imaging markers for pediatric idiopathic intracranial hypertension: a retrospective study
Source: Pediatr Radiol. 2026 May 23;56(7):1516–35. doi: 10.1007/s00247-026-06638-7 (PMC13357526; doi:10.1007/s00247-026-06638-7)
Supplement: Supplementary file 1 — (DOCX 228 KB) [file 247_2026_6638_MOESM1_ESM.docx]

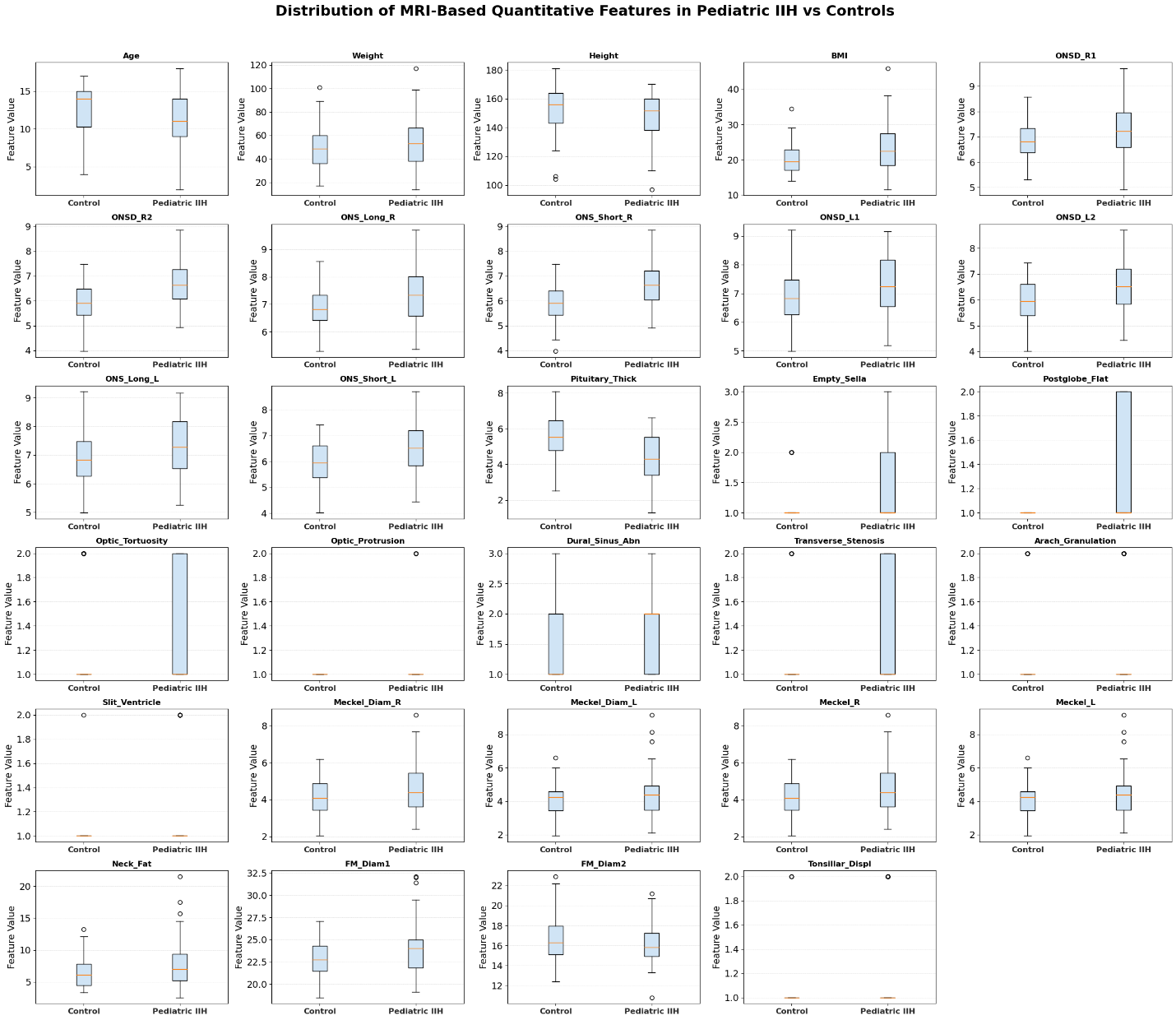


**Fig. 1** Group-wise boxplots illustrating the distribution of magnetic resonance imaging features in pediatric idiopathic intracranial hypertension (Class 1) versus control participants (Class 0)

Each boxplot summarizes the median, interquartile range, and spread of one feature in the headache control group and the pediatric idiopathic intracranial hypertension group. Variables showing the clearest separation include optic nerve sheath measurements, empty sella degree, and posterior globe flattening. *MRI* magnetic resonance imaging; *IIH* idiopathic intracranial hypertension
