# Supplementary material for: Diagnostic value of machine-learning using conventional magnetic resonance imaging markers for pediatric idiopathic intracranial hypertension: a retrospective study
Source: Pediatr Radiol. 2026 May 23;56(7):1516–35. doi: 10.1007/s00247-026-06638-7 (PMC13357526; doi:10.1007/s00247-026-06638-7)
Supplement: Supplementary file 2 — (DOCX 22.8 KB) [file 247_2026_6638_MOESM2_ESM.docx]

**Table 1** Common Optuna workflow settings used across all six classifiers

The table summarizes the shared Bayesian hyperparameter-optimization settings used in each repetition of the repeated nested machine-learning workflow, including the sampler, pruning strategy, split structure, feature-selection method, preprocessing, and optimization target. The outer split reserved 20% of the cohort for independent testing, while the remaining data were divided into inner training and validation subsets for model selection. *TPE* Tree-structured Parzen Estimator, *ANOVA* analysis of variance

| **Item** | **Setting** | **Notes** |
| --- | --- | --- |
| Optuna sampler | Tree-structured Parzen Estimator (TPE) | Bayesian sampler |
| Pruner | Median Pruner | Warm-up = 10 trials; prunes trials below the running median |
| Trials per model | 150 | Default |
| Repetitions | 20 | Same repeated evaluation framework applied across models |
| Split strategy | StratifiedShuffleSplit | Outer test = 20%; inner train ≈ 65%, validation ≈ 15% |
| Positive class | CLASS = 1 (class1) | Metrics computed with respect to the positive class |
| Feature selector | SelectKBest | ANOVA F-statistic; k = 3–15, clipped to the number of available features |
| Data preprocessing | Robust scaling | Applied before feature selection and model fitting |
| Hyperparameter optimization target | F1-score of the positive class | Computed on the inner validation split |
| Reproducibility | rng_seed | Reused across repetitions where supported |

TPE, Tree-structured Parzen Estimator.
Split description: the same outer and inner data-partitioning logic was reused across models within each repetition.

**Table 2** Model-specific hyperparameter search spaces used in the Optuna workflow The table lists the candidate hyperparameters explored for each classifier during Optuna-based Bayesian optimization, together with the search ranges/options and implementation notes. The same repeated outer/inner split structure was applied across models, and 150 optimization trials were performed per model within each repetition. *SVM* support vector machine, *MLP* multilayer perceptron, *KNN* k-nearest neighbors, *XGBoost* extreme gradient boosting

| **Model** | **Hyperparameter** | **Search space / options** | **Notes** |
| --- | --- | --- | --- |
| Random Forest | n_estimators | 200 to 2000 (step 200) | Number of trees |
| Random Forest | max_depth | 2 to 80 | Maximum depth of each tree |
| Random Forest | min_samples_split | 2 to 50 | Minimum samples to split an internal node |
| Random Forest | min_samples_leaf | 1 to 20 | Minimum samples per leaf |
| Random Forest | max_features | sqrt, log2, None, 0.3, 0.5, 0.7 | Mix of categorical and fractional feature subsets |
| Random Forest | criterion | gini, entropy, log_loss | Split criterion |
| Random Forest | bootstrap | True, False | Use bootstrap samples |
| SVM | kernel | linear, rbf, poly, sigmoid | Chooses SVC kernel |
| SVM | C | 1e-4 to 1e4 (log-uniform) | Regularization strength |
| SVM | gamma_mode | scale, auto, float | If float, a numeric gamma is sampled |
| SVM | gamma | 1e-6 to 10.0 (log-uniform) | Only if gamma_mode = float and kernel is rbf, poly, or sigmoid |
| SVM | degree | 2 to 7 | Only if kernel = poly |
| SVM | coef0 | -5 to 5 (uniform) | Only if kernel = poly or sigmoid |
| MLP | hidden_layer_sizes | (50,), (100,), (150,), (50,25), (100,50), (150,75), (100,100), (150,100), (200,100,50), (300,150,75) | Fixed candidate **topologies** |
| MLP | activation | relu, tanh, logistic | Hidden activation |
| MLP | alpha | 1e-6 to 1e-1 (log-uniform) | L2 penalty |
| MLP | solver | adam, lbfgs | Optimizer |
| MLP | max_iter | 300 to 3000 (step 300) | Maximum training iterations |
| MLP | learning_rate | constant, adaptive | Only if solver = adam |
| MLP | learning_rate_init | 1e-6 to 5e-3 (log-uniform) | Only if solver = adam |
| MLP | beta_1 | 0.5 to 0.99 (uniform) | Adam β1 |
| MLP | beta_2 | 0.8 to 0.999 (uniform) | Adam β2 |
| MLP | batch_size | 16, 32, 64, 128 | Only if solver = adam |
| MLP | early_stopping | True, False | Only if solver = adam |
| XGBoost | n_estimators | 300 to 3000 (step 300) | Number of boosting rounds |
| XGBoost | max_depth | 2 to 20 | Maximum tree depth |
| XGBoost | learning_rate | 1e-4 to 0.3 (log-uniform) | Shrinkage |
| XGBoost | subsample | 0.3 to 1.0 (uniform) | Row subsampling |
| XGBoost | colsample_bytree | 0.3 to 1.0 (uniform) | Column subsampling per tree |
| XGBoost | min_child_weight | 1e-3 to 50 (log-uniform) | Minimum Hessian weight in a child |
| XGBoost | gamma | 0.0 to 10.0 (uniform) | Minimum loss reduction to split |
| XGBoost | reg_alpha | 1e-8 to 10.0 (log-uniform) | L1 regularization |
| XGBoost | reg_lambda | 1e-3 to 10.0 (log-uniform) | L2 regularization |
| XGBoost | scale_pos_weight | 0.5 to 3.0 (uniform) | Class imbalance reweighting factor |
| KNN | n_neighbors | 1 to safe_k | safe_k = min(15, len(X_train) − 1) |
| KNN | weights | uniform, distance | Distance weighting |
| KNN | p | 1 to 5 | Minkowski order (p = 1 Manhattan, p = 2 Euclidean, etc.) |
| KNN | leaf_size | 10 to 100 | Tree-based search leaf size |
| KNN | metric | minkowski, euclidean, manhattan, chebyshev | Distance metric |
| Bagging | base_estimator | tree, knn | Chooses tree-based versus KNN-based base learner |
| Bagging | n_estimators | 50 to 500 (step 50) | Number of base estimators |
| Bagging | max_samples | 0.3 to 1.0 (uniform) | Fraction of samples per estimator |
| Bagging | max_features | 0.3 to 1.0 (uniform) | Fraction of features per estimator |
| Bagging | bootstrap | True, False | Bootstrap samples |
| Bagging | bootstrap_features | False, True | Bootstrap features |
| Bagging–Tree base | max_depth | 1 to 50 | Maximum depth of base trees |
| Bagging–Tree base | min_samples_split | 2 to 50 | Minimum samples to split |
| Bagging–Tree base | min_samples_leaf | 1 to 20 | Minimum samples per leaf |
| Bagging–KNN base | n_neighbors | 1 to safe_k_bag | safe_k_bag = min(10, len(X_train) − 1) |
| Bagging–KNN base | weights | uniform, distance | Same as standalone KNN |

*random_state = rng_seed* was used for reproducibility where supported. Fixed settings were as follows: Random forest, *class_weight* = balanced and *n_jobs* = -1, SVM, *class_weight* = balanced and probability = True, MLP, random_state = rng_seed, XGBoost, tree_method = hist, eval_metric = logloss, n_jobs = -1, and random_state = rng_seed, Bagging, n_jobs = -1 and random_state = rng_seed, Bagging–Tree base learner, class_weight = balanced and random_state = rng_seed. For SVM, gamma was sampled only when gamma_mode = float and the kernel was rbf, poly, or sigmoid. For MLP, learning_rate, learning_rate_init, beta_1, beta_2, batch_size, and early_stopping were sampled only when solver = adam. SVM, support vector machine, MLP, multilayer perceptron, KNN, k-nearest neighbors
