# Supplementary material for: Diagnostic value of machine-learning using conventional magnetic resonance imaging markers for pediatric idiopathic intracranial hypertension: a retrospective study
Source: Pediatr Radiol. 2026 May 23;56(7):1516–35. doi: 10.1007/s00247-026-06638-7 (PMC13357526; doi:10.1007/s00247-026-06638-7)
Supplement: Supplementary file 3 — (DOCX 17.4 KB) [file 247_2026_6638_MOESM3_ESM.docx]

CONSTANTS:

N_REPS = 20

TEST_FRACTION = 0.20 # outer test set

VAL_FRACTION_GLOBAL = 0.15 # global validation fraction

TRAIN_IN_TRAINVAL = 0.8125 # ≈65% of full data

VAL_IN_TRAINVAL = 0.1875 # ≈15% of full data

MODELS = {Random Forest, SVM, MLP, XG-Boost, KNN, Bagging}

N_TRIALS_PER_MODEL = 150

K_MIN = 3, K_MAX = 15 # number of selected features

OPTIMIZE_FOR = F1-score # primary objective

POSITIVE_CLASS = "Pediatric IIH" (label = 1)

------------------------------------------------------------

STEP 1: LOAD DATA AND DEFINE TARGET/FEATURES

------------------------------------------------------------

Read Excel file into Data Frame DF

y = first column # Group (0/1)

X = remaining columns # Feature space

-----------------------------------------------------------

STEP 2: MAIN OUTER LOOP (REPEATED N_REPS TIMES)

------------------------------------------------------------

FOR rep in {1, ..., N_REPS} DO

# ---- Outer stratified split: train+val vs test ----

(trainval_idx, test_idx): = StratifiedShuffleSplit (X, y, test size = TEST_FRACTION)

X_trainval = X[trainval_idx]

y_trainval = y[trainval_idx]

X_test= X[test_idx]

y_test = y[test_idx]

# ---- Inner stratified split: train vs validation ----

(train_idx, val_idx): = StratifiedShuffleSplit (X_trainval, y_trainval, train_size =

TRAIN_IN_TRAINVAL, test_size = VAL_IN_TRAINVAL)

X_train = X_trainval[train_idx]

y_train = y_trainval[train_idx]

X_val = X_trainval[val_idx]

y_val = y_trainval[val_idx]

# ---- Preprocessor (Robust-Scaler on all features) ----

PREPROC=Column-Transformer (apply Robust-Scaler to all feature in X_train)

--------------------------------------------------------

STEP 2a: MODEL-SPECIFIC OPTIMIZATION (INNER LOOP)

--------------------------------------------------------

FOR each model_name in MODELS DO

# Create Optuna study to maximize validation F1-score

STUDY= Optuna create study (direction = "maximize")

# Objective function for one Optuna trial

DEFINE objective(trial):

# ---- Sample classifier hyperparameters ----

clf_params = sample model specific hyperparameters (model_name, trial)

# ---- Sample number of selected features k ----

n_features= number_of_features(X_train)

k_best= trial. suggest_int ("k_best", K_MIN, min (K_MAX, n_features, 15))

# ---- Define feature selector ----

FEAT_SELECTOR= SelectKBest (score_func = ANOVA_F_test (f_classif), k = k_best)

# ---- Build pipeline: PREPROC + FEAT_SELECTOR + classifier ----

clf = instantiate_classifier (model_name, clf_params)

PIPE = Pipeline (steps = [("prep", PREPROC), ("feat", FEAT_SELECTOR), ("clf", clf)])

# ---- Fit on training set ----

PIPE.fit (X_train, y_train)

# ---- Predict on validation set ----

y_val_pred = PIPE.predict(X_val)

y_val_proba= PIPE.predict_proba(X_val) [:, class = 1]

# ---- Compute validation metrics ----

F1_val= F1_score (y_val, y_val_pred, positive_class = 1)

Average_Precision_val = Average_Precision (y_val, y_val_proba)

AUC_val = ROC_AUC (y_val, y_val_proba)

# ---- Store additional metrics for logging ----

trial.user_attrs["val_f1"] = F1_val

trial.user_attrs["val_average_precision "] = Average_Precision_val

trial.user_attrs["val_auc"] = AUC_val

RETURN F1_val # optimization target

# ---- Run Optuna search for this model ----

STUDY.optimize(objective, n_trials = N_TRIALS_PER_MODEL)

# ---- Extract best hyperparameters and k ----

best_params = STUDY.best_trial. params

best_k = best_params["k_best"]

# ---- Sanitize parameters (safe KNN, safe Bagging etc.) ----

clean_params, base_choice = sanitize_params_for_final (model_name, best_params)

# ---- Build final classifier with best hyperparameters ----

final_clf = build_final_classifier (model_name, clean_params, base_choice)

# ---- Build final pipeline and train on full TRAIN+VAL ----

FINAL_FEAT_SELECTOR= SelectKBest (score_func = ANOVA_F_test, k = best_k)

FINAL_PIPE = Pipeline (steps = [("prep", PREPROC),"feat", FINAL_FEAT_SELECTOR), ("clf", final_clf)])

FINAL_PIPE.fit (X_trainval, y_trainval)

# ---- Store selected feature names for this run/model ----

selected_features = get_selected_features (FINAL_PIPE, X)

# ---- Evaluate on independent TEST set ----

y_test_pred = FINAL_PIPE.predict(X_test)

y_test_proba = FINAL_PIPE.predict_proba(X_test) [:, class = 1]

cm_test = confusion_matrix (y_test, y_test_pred, labels = {0,1})

F1_test = F1_score (y_test, y_test_pred, positive_class = 1)

AUC_test = ROC_AUC (y_test, y_test_proba)

Average_Precision _test = Average_Precision (y_test, y_test_proba)

# ---- Store metrics and artifacts ----

store_metrics (rep, model_name, F1_test, AUC_test, Average_Precision _test, cm_test, STUDY.best_trial.user_attrs, selected_features)

save_trained_model (FINAL_PIPE, rep, model_name, F1_test, AUC_test, Average_Precision _test)

END FOR # end loop over MODELS

END FOR # end outer loop over N_REPS

**Supplementary Material** Stepwise pseudocode of the supervised machine-learning pipeline used to distinguish pediatric idiopathic intracranial hypertension from headache controls

This pseudocode summarizes the full repeated nested workflow used in the study: loading the tabular feature matrix, performing 20 stratified outer repetitions, splitting the data into training, validation, and independent test subsets, applying robust scaling and univariate analysis-of-variance feature selection, tuning six classifiers with Optuna, retraining the best configuration on the combined training and validation data, and evaluating final performance on the held-out test set *IIH* idiopathic intracranial hypertension, *SVM* support vector machine, *MLP* multilayer perceptron, *KNN* k-nearest neighbors, *ANOVA* analysis of variance, *AUC* area under the receiver operating characteristic curve
