# Supplementary material for: Diagnostic value of machine-learning using conventional magnetic resonance imaging markers for pediatric idiopathic intracranial hypertension: a retrospective study
Source: Pediatr Radiol. 2026 May 23;56(7):1516–35. doi: 10.1007/s00247-026-06638-7 (PMC13357526; doi:10.1007/s00247-026-06638-7)
Supplement: Supplementary file 4 — (DOCX 116 KB) [file 247_2026_6638_MOESM4_ESM.docx]

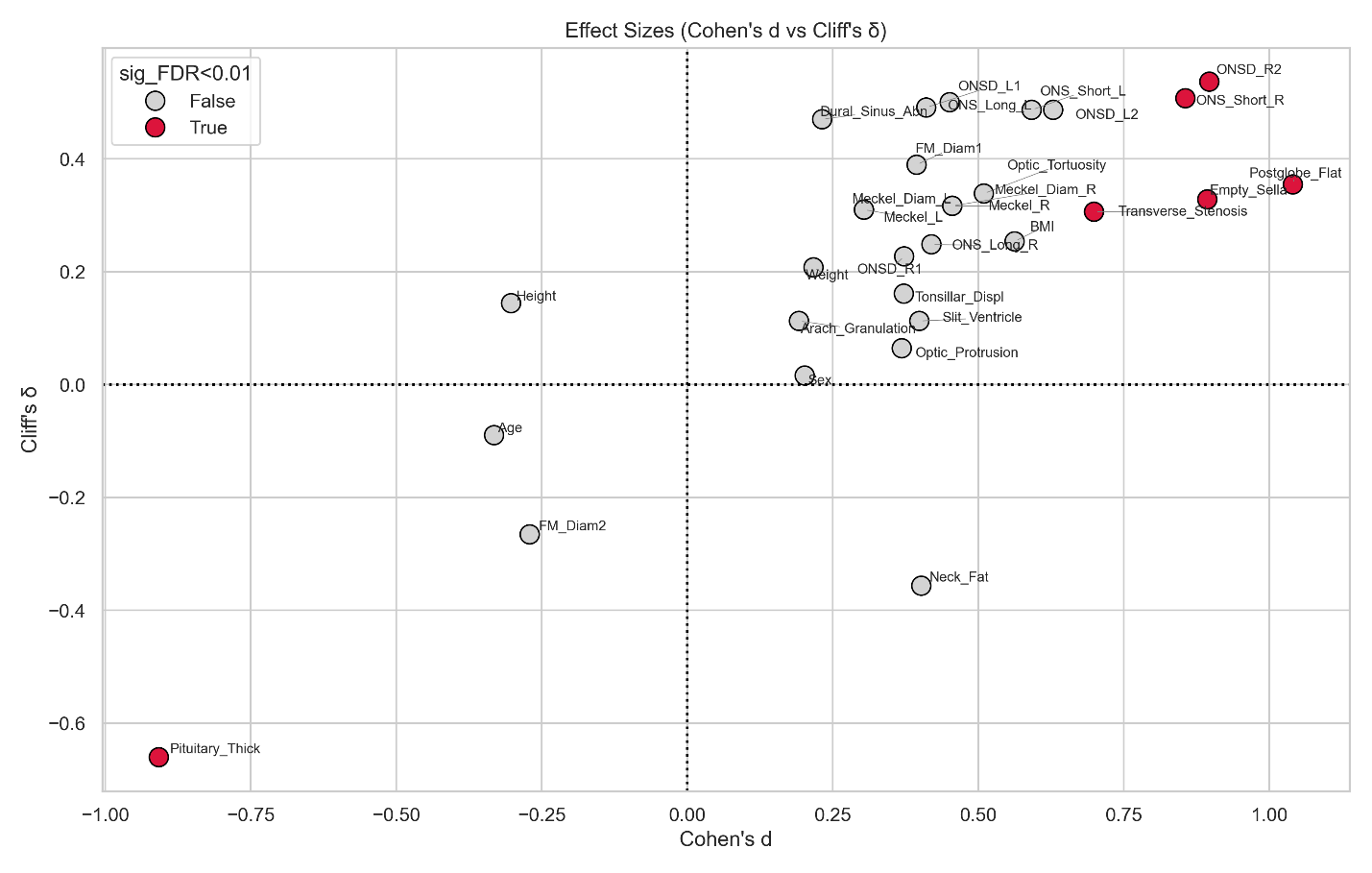


**Fig.1** Effect size distribution of magnetic resonance imaging-derived Features based on Cohen’s d and Cliff’s δ. *BMI* body mass index, *Dural_Sinus_Abn* dural venous sinus abnormality, *FM_Diam* foramen magnum diameter, *Meckel_Diam*, Meckel’s cave diameter, *Neck_Fat* neck fat tissue thickness, *ONSD* optic nerve sheath diameter, *Pituitary_Thick* pituitary gland thickness, *Postglobe_Flat* posterior globe flattening, *Transverse_Stenosis* transverse venous sinus stenosis, *Tonsillar_Displ* inferior tonsillar displacement, *Arach_Granulation* arachnoid granulation, *Slit_Ventricle* slit ventricle, *Optic_Protrusion* optic nerve protrusion, *Optic_Tortuosity* optic nerve tortuosity, *Empty_Sella* empty sella, *sig_FDR<0.01* significant after false discovery rate correction at P<0.01

The figure summarizes the magnitude and direction of between-group differences for all evaluated features using both parametric (Cohen’s d) and non-parametric (Cliff’s delta) effect-size measures. Larger absolute values indicate stronger separation between pediatric idiopathic intracranial hypertension and headache controls; the strongest effects were observed for right optic nerve sheath short axis, right optic nerve sheath diameter in the coronal plane, transverse venous sinus stenosis, and empty sella.
