# Supplementary material for: Diagnostic value of machine-learning using conventional magnetic resonance imaging markers for pediatric idiopathic intracranial hypertension: a retrospective study
Source: Pediatr Radiol. 2026 May 23;56(7):1516–35. doi: 10.1007/s00247-026-06638-7 (PMC13357526; doi:10.1007/s00247-026-06638-7)
Supplement: Supplementary file 5 — (DOCX 111 KB) [file 247_2026_6638_MOESM5_ESM.docx]

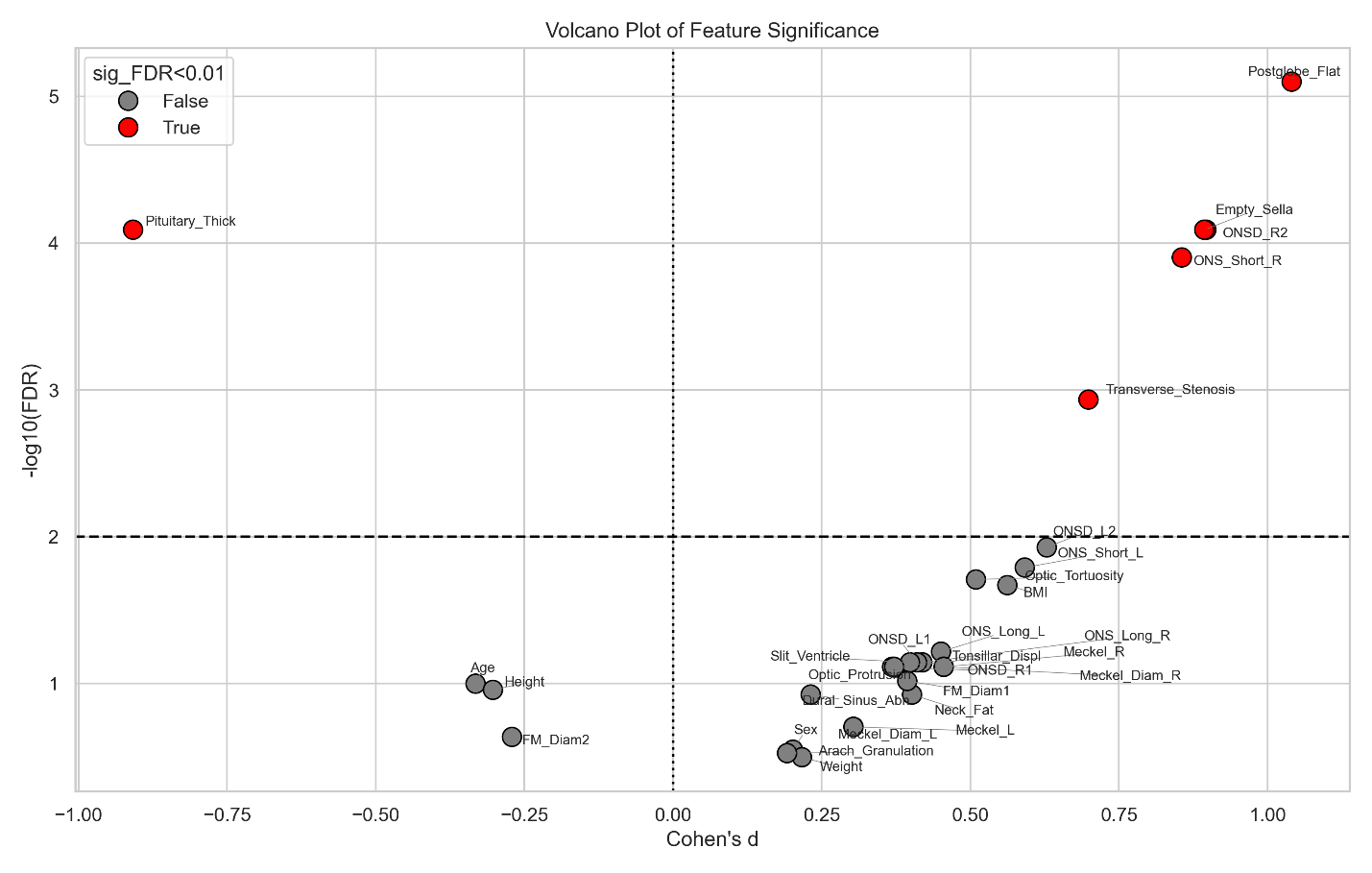


**Fig. 1** Volcano plot of statistical significance and effect size for magnetic resonance imaging-derived features *FDR* false discovery rate, *MRI*, magnetic resonance imaging

This volcano plot combines effect size and statistical significance for all evaluated features. Features above the false-discovery-rate threshold and with larger effect sizes provide the strongest separation between pediatric idiopathic intracranial hypertension and headache controls; posterior globe flattening, empty sella, right optic nerve sheath short axis, and transverse venous sinus stenosis are the clearest examples.
