# Supplementary material for: Diagnostic value of machine-learning using conventional magnetic resonance imaging markers for pediatric idiopathic intracranial hypertension: a retrospective study
Source: Pediatr Radiol. 2026 May 23;56(7):1516–35. doi: 10.1007/s00247-026-06638-7 (PMC13357526; doi:10.1007/s00247-026-06638-7)
Supplement: Supplementary file 6 — (DOCX 0.99 MB) [file 247_2026_6638_MOESM6_ESM.docx]

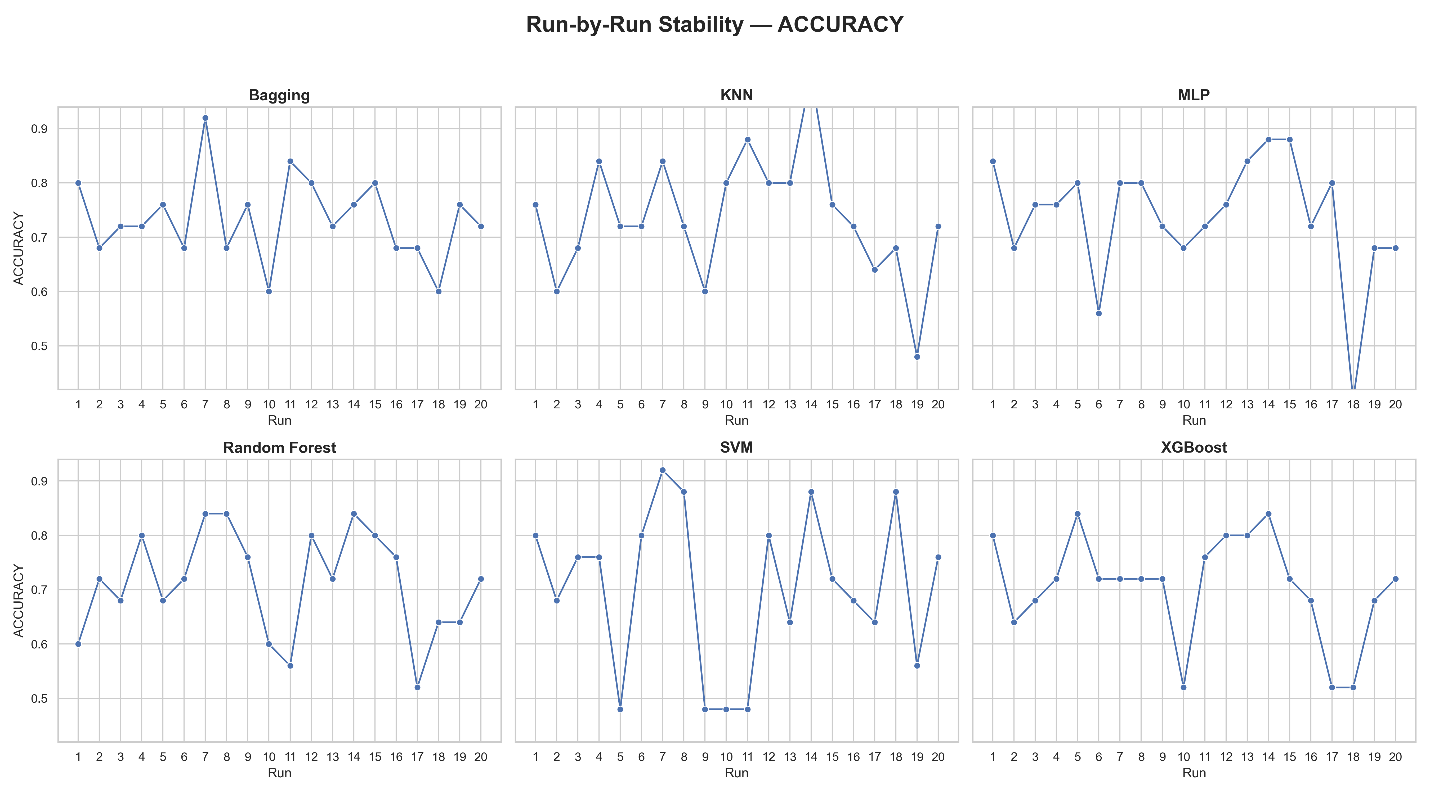

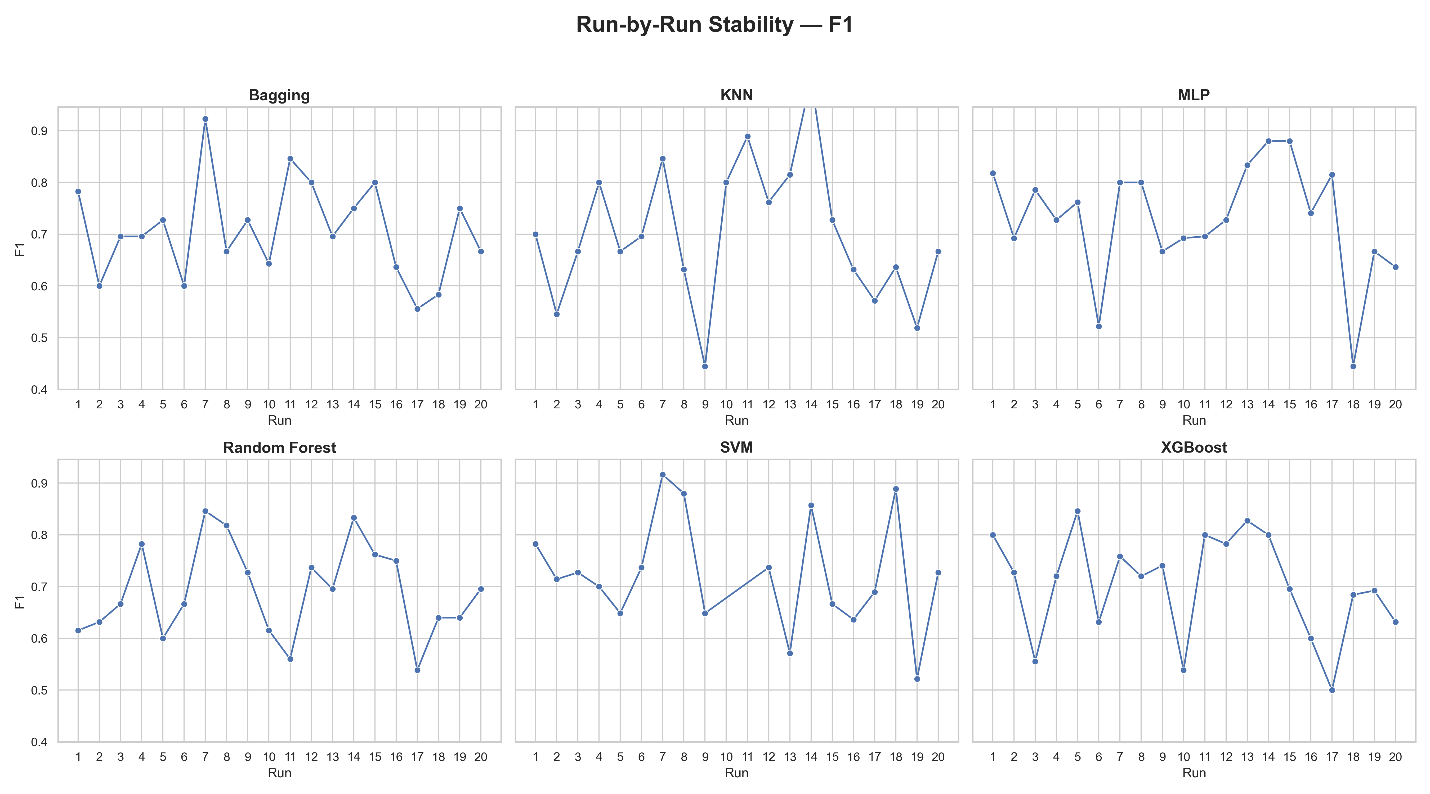

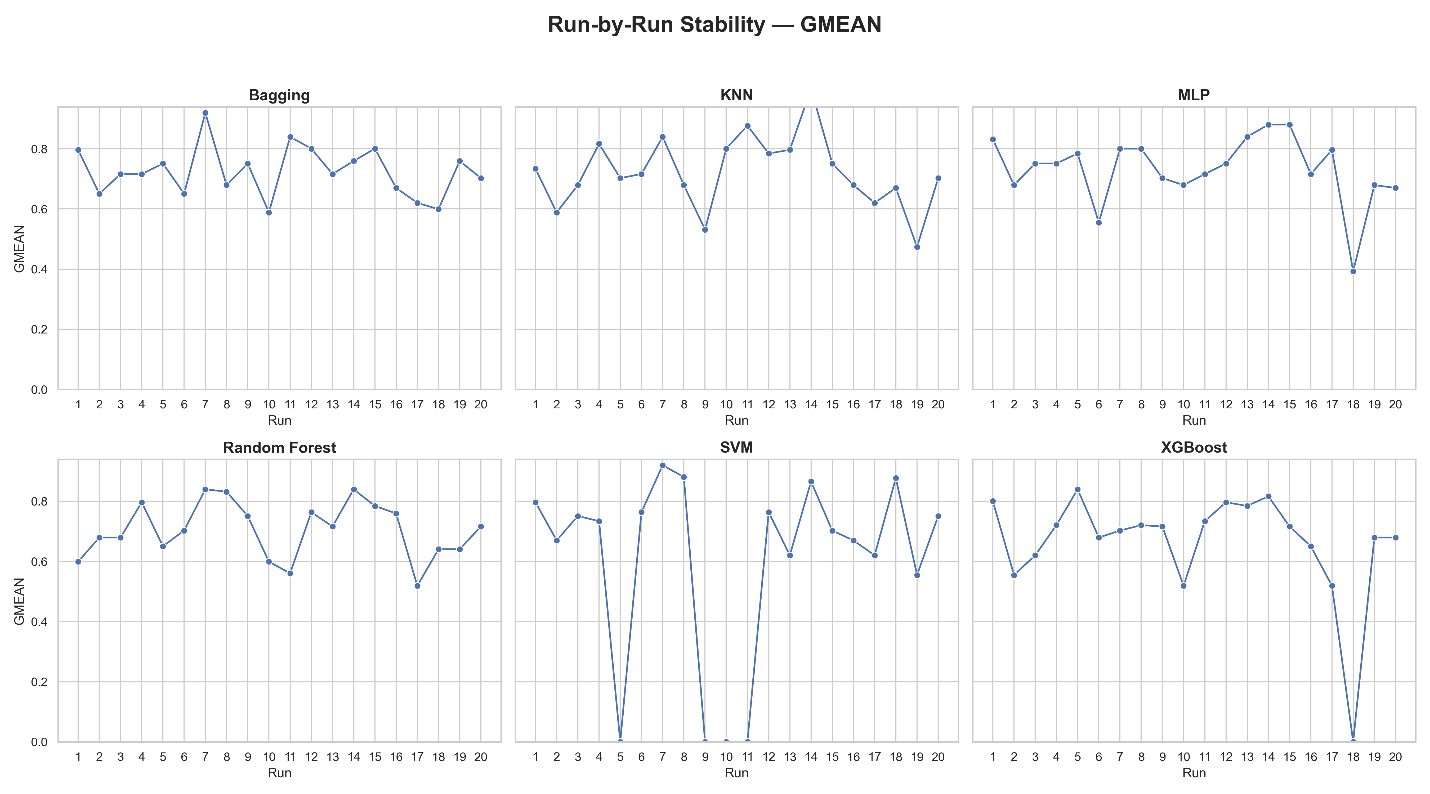

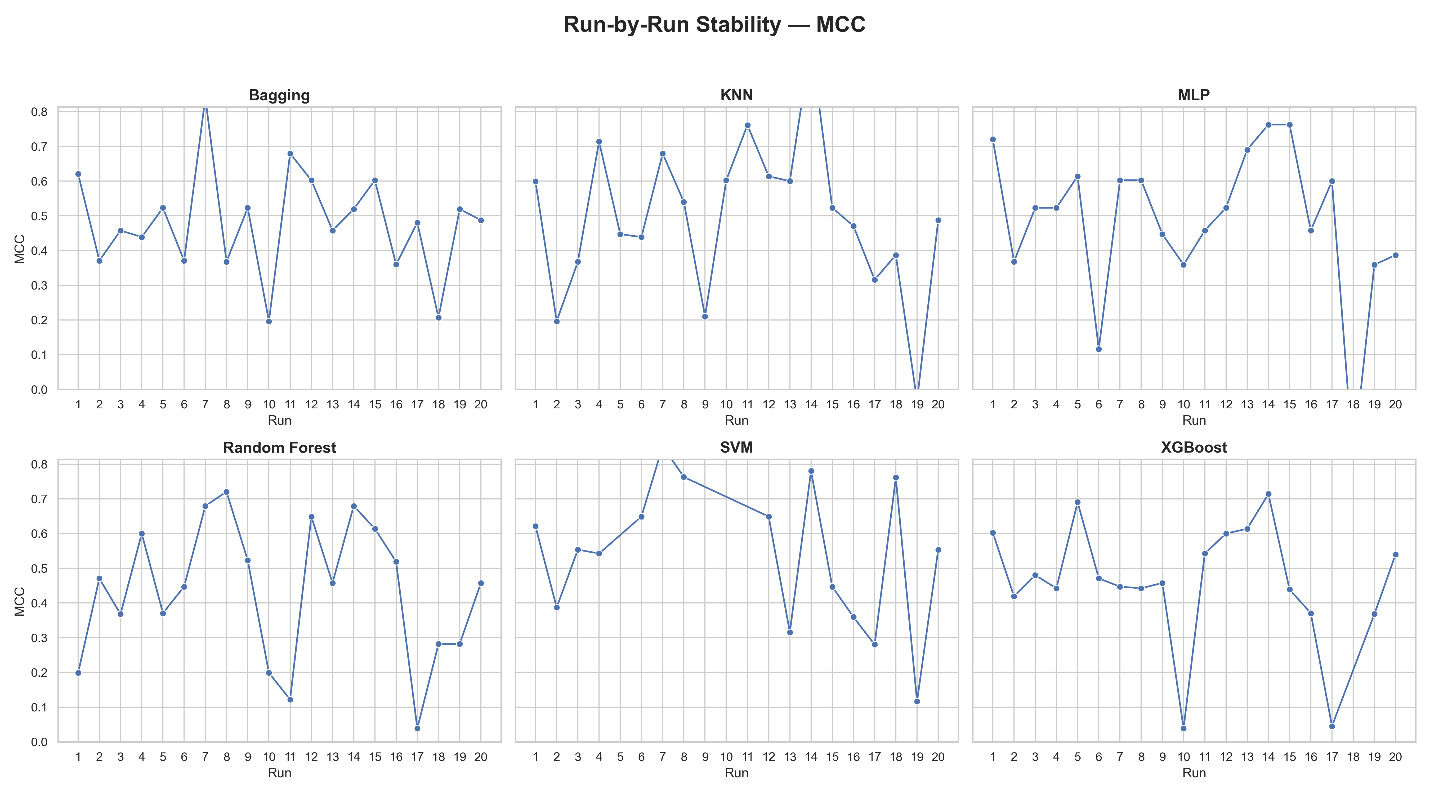

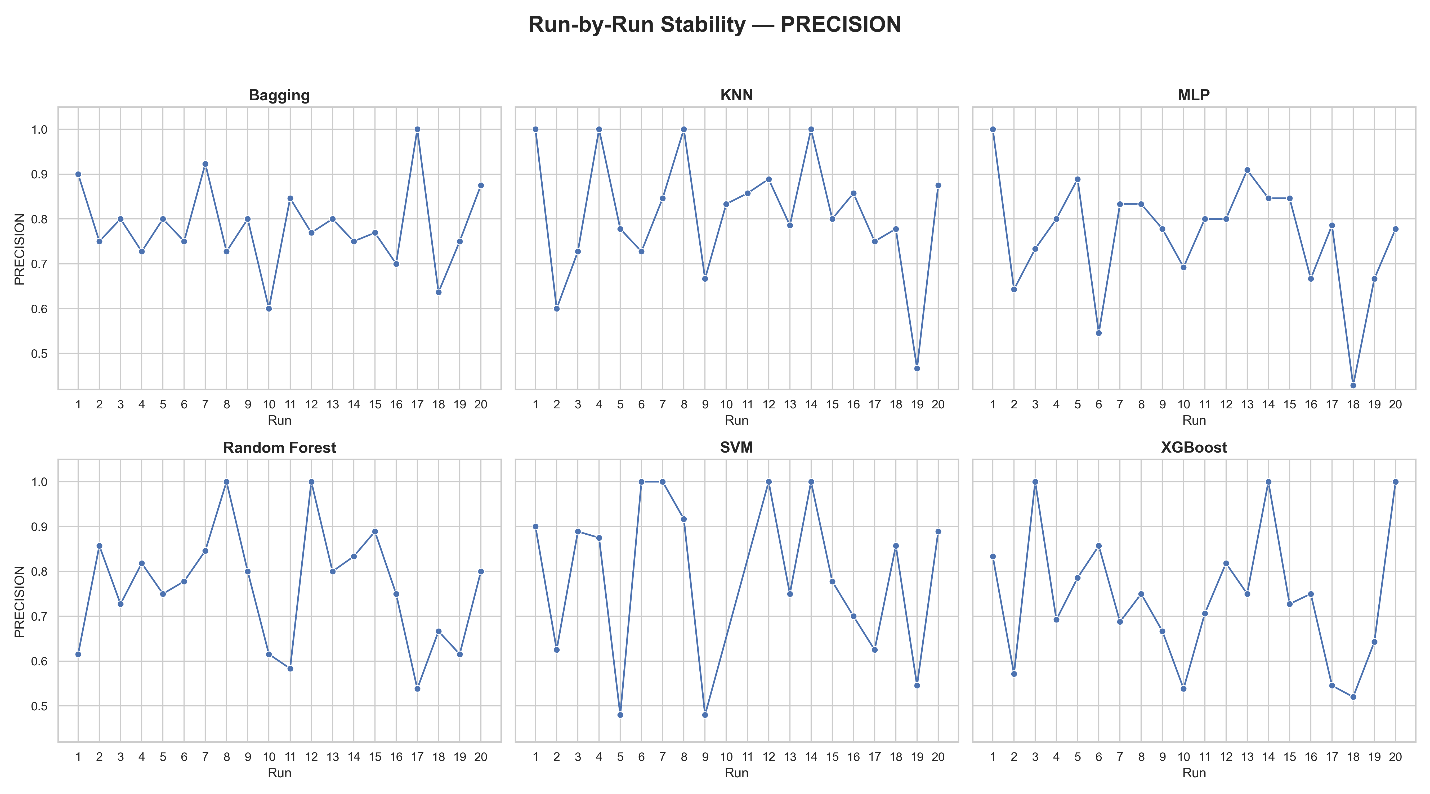

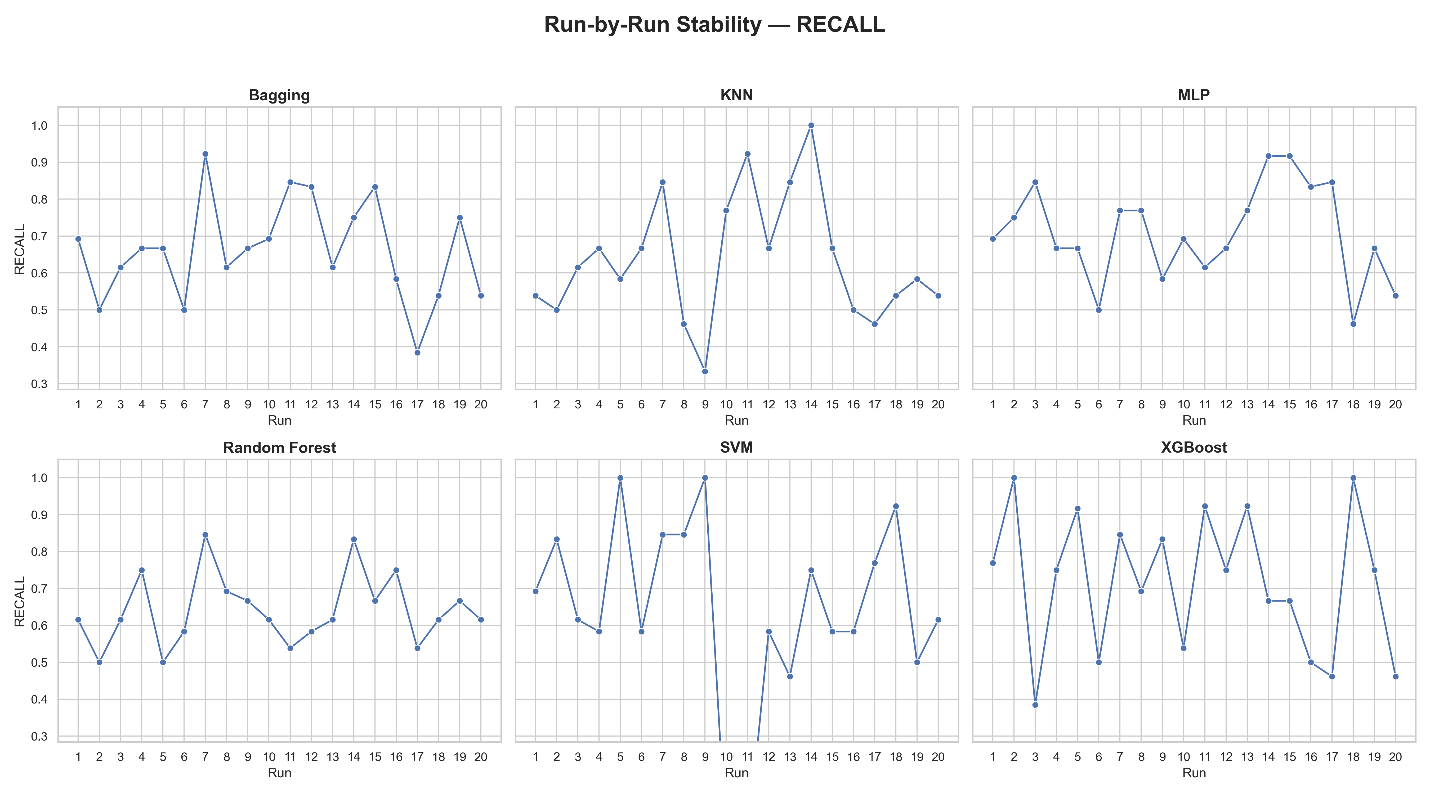

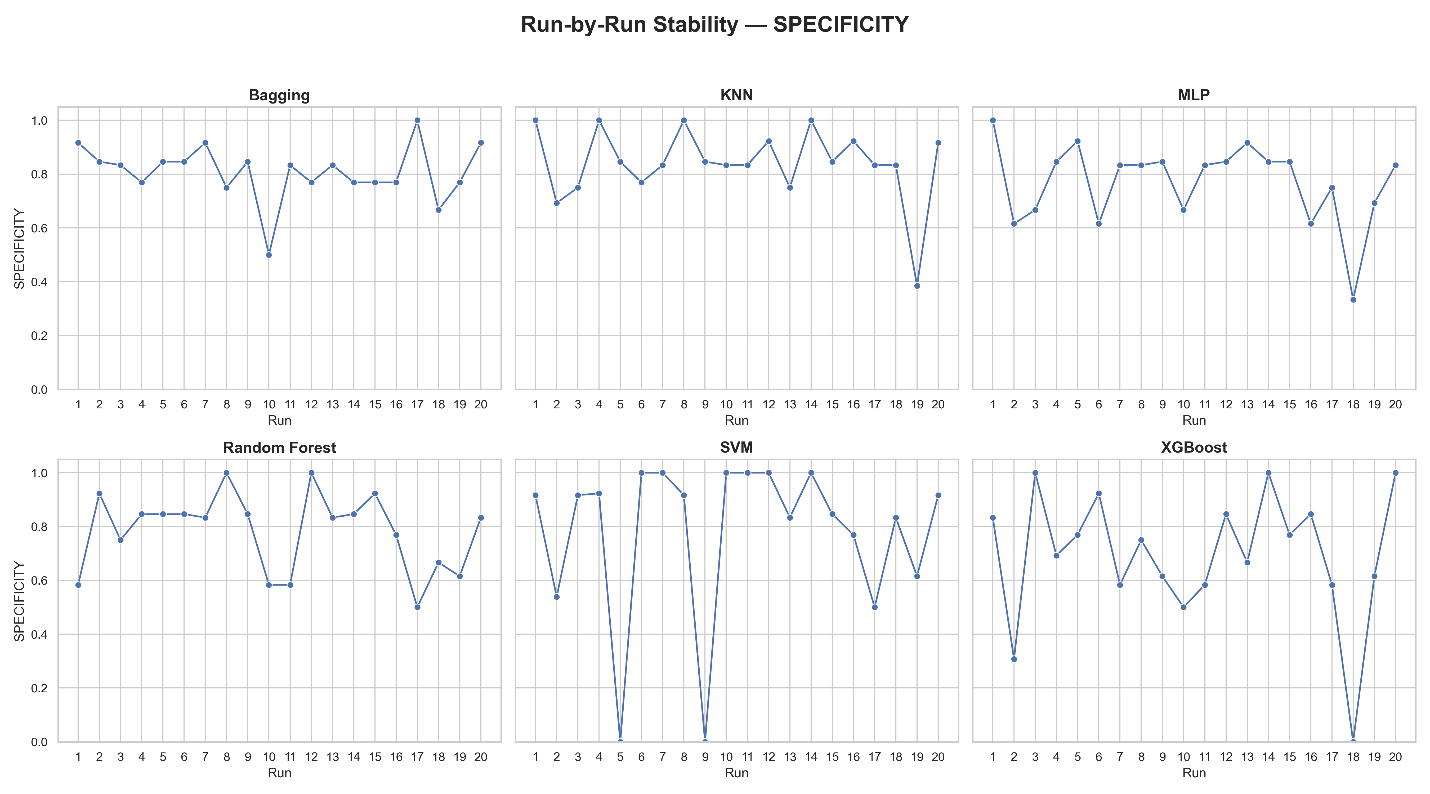


**Fig. 1** Run‑by‑run stability of classification performance across 20 independent repetitions

This figure shows the trajectories of accuracy, F1-score, geometric mean, Matthews correlation coefficient, precision, recall, and specificity across 20 repeated test evaluations for all six classifiers. Bagging, k-nearest neighbors, multilayer perceptron, and random forest fluctuate within relatively narrow bands, whereas extreme gradient boosting and especially support vector machine show greater fold-to-fold variability. *KNN* k-nearest neighbors; *MLP* multilayer perceptron; *MCC* Matthews correlation coefficient; *SVM* support vector machine; *XGBoost* extreme gradient boosting
