# Supplementary material for: Diagnostic value of machine-learning using conventional magnetic resonance imaging markers for pediatric idiopathic intracranial hypertension: a retrospective study
Source: Pediatr Radiol. 2026 May 23;56(7):1516–35. doi: 10.1007/s00247-026-06638-7 (PMC13357526; doi:10.1007/s00247-026-06638-7)
Supplement: Supplementary file 7 — (DOCX 29.4 KB) [file 247_2026_6638_MOESM7_ESM.docx]

**Table 1** Pairwise Wilcoxon signed-rank tests with Holm adjustment across classifiers for all evaluation metrics.

The table summarizes pairwise comparisons of classifier performance across repeated test evaluations. For each metric, the table reports the difference in mean percentage points, the unadjusted Wilcoxon signed-rank *P* value, and the Holm-adjusted *P* value. No pairwise comparison remained statistically significant after correction for multiple testing. *G-mean* geometric mean of sensitivity and specificity, *MCC* Matthews correlation coefficient, *AUC* area under the receiver operating characteristic curve, *PR-AUC* area under the precision-recall curve, *ns* not significant

| **Metric** | **Comparison** | **Δ Mean points** | **Wilcoxon *P*** | **Holm-adjusted *P*** | **Significance** |
| --- | --- | --- | --- | --- | --- |
| Accuracy | Bagging vs. KNN | -0.400 | 0.965 | 1 | ns |
|  | Bagging vs. MLP | -0.400 | 0.761 | 1 | ns |
|  | Bagging vs. Random Forest | 2.200 | 0.413 | 1 | ns |
|  | Bagging vs. SVM | 3.000 | 0.459 | 1 | ns |
|  | Bagging vs. XGBoost | 2.800 | 0.129 | 1 | ns |
|  | KNN vs. MLP | 0.000 | 0.824 | 1 | ns |
|  | KNN vs. Random Forest | 2.600 | 0.459 | 1 | ns |
|  | KNN vs. SVM | 3.400 | 0.616 | 1 | ns |
|  | KNN vs. XGBoost | 3.200 | 0.241 | 1 | ns |
|  | MLP vs. Random Forest | 2.600 | 0.277 | 1 | ns |
|  | MLP vs. SVM | 3.400 | 0.288 | 1 | ns |
|  | MLP vs. XGBoost | 3.200 | 0.131 | 1 | ns |
|  | Random Forest vs. SVM | 0.800 | 0.840 | 1 | ns |
|  | Random Forest vs. XGBoost | 0.600 | 0.753 | 1 | ns |
|  | SVM vs. XGBoost | -0.200 | 0.856 | 1 | ns |
|  |  |  |  |  |  |
|  |  |  |  |  |  |
| **Metric** | **Comparison** | **Δ Mean points** | **Wilcoxon *P*** | **Holm-adjusted *P*** | **Significance** |
| G-MEAN | Bagging vs. KNN | 0.225 | 0.906 | 1 | ns |
|  | Bagging vs. MLP | -0.855 | 0.701 | 1 | ns |
|  | Bagging vs. Random Forest | 2.089 | 0.528 | 1 | ns |
|  | Bagging vs. SVM | 12.732 | 0.352 | 1 | ns |
|  | Bagging vs. XGBoost | 6.193 | 0.048 | 0.678 | ns |
|  | KNN vs. MLP | -1.080 | 0.522 | 1 | ns |
|  | KNN vs. Random Forest | 1.864 | 0.528 | 1 | ns |
|  | KNN vs. SVM | 12.506 | 0.457 | 1 | ns |
|  | KNN vs. XGBoost | 5.967 | 0.177 | 1 | ns |
|  | MLP vs. Random Forest | 2.944 | 0.314 | 1 | ns |
|  | MLP vs. SVM | 13.587 | 0.099 | 1 | ns |
|  | MLP vs. XGBoost | 7.048 | 0.013 | 0.189 | ns |
|  | Random Forest vs. SVM | 10.642 | 0.334 | 1 | ns |
|  | Random Forest vs. XGBoost | 4.104 | 0.277 | 1 | ns |
|  | SVM vs. XGBoost | -6.539 | 0.985 | 1 | ns |
|  |  |  |  |  |  |
|  |  |  |  |  |  |
| **Metric** | **Comparison** | **Δ Mean points** | **Wilcoxon *P*** | **Holm-adjusted *P*** | **Significance** |
| MCC | Bagging vs. KNN | -0.123 | 0.975 | 1 | ns |
|  | Bagging vs. MLP | -3.354 | 0.561 | 1 | ns |
|  | Bagging vs. Random Forest | 2.088 | 0.950 | 1 | ns |
|  | Bagging vs. SVM | -2.500 | 0.552 | 1 | ns |
|  | Bagging vs. XGBoost | 3.334 | 0.847 | 1 | ns |
|  | KNN vs. MLP | -3.232 | 0.599 | 1 | ns |
|  | KNN vs. Random Forest | 2.211 | 0.753 | 1 | ns |
|  | KNN vs. SVM | -2.378 | 0.599 | 1 | ns |
|  | KNN vs. XGBoost | 3.457 | 0.489 | 1 | ns |
|  | MLP vs. Random Forest | 5.443 | 0.561 | 1 | ns |
|  | MLP vs. SVM | 0.854 | 1.000 | 1 | ns |
|  | MLP vs. XGBoost | 6.689 | 0.169 | 1 | ns |
|  | Random Forest vs. SVM | -4.588 | 0.272 | 1 | ns |
|  | Random Forest vs. XGBoost | 1.246 | 0.679 | 1 | ns |
|  | SVM vs. XGBoost | 5.834 | 0.121 | 1 | ns |
|  |  |  |  |  |  |
|  |  |  |  |  |  |
| **Metric** | **Comparison** | **Δ Mean points** | **Wilcoxon *P*** | **Holm-adjusted *P*** | **Significance** |
| Precision | Bagging vs. KNN | -1.772 | 0.670 | 1 | ns |
|  | Bagging vs. MLP | 2.476 | 0.551 | 1 | ns |
|  | Bagging vs. Random Forest | 0.793 | 0.856 | 1 | ns |
|  | Bagging vs. SVM | -0.458 | 0.796 | 1 | ns |
|  | Bagging vs. XGBoost | 3.498 | 0.442 | 1 | ns |
|  | KNN vs. MLP | 4.248 | 0.320 | 1 | ns |
|  | KNN vs. Random Forest | 2.565 | 0.551 | 1 | ns |
|  | KNN vs. SVM | 1.314 | 0.619 | 1 | ns |
|  | KNN vs. XGBoost | 5.269 | 0.179 | 1 | ns |
|  | MLP vs. Random Forest | -1.683 | 0.442 | 1 | ns |
|  | MLP vs. SVM | -2.934 | 0.580 | 1 | ns |
|  | MLP vs. XGBoost | 1.022 | 0.640 | 1 | ns |
|  | Random Forest vs. SVM | -1.251 | 0.619 | 1 | ns |
|  | Random Forest vs. XGBoost | 2.704 | 0.586 | 1 | ns |
|  | SVM vs. XGBoost | 3.955 | 0.326 | 1 | ns |
|  |  |  |  |  |  |
|  |  |  |  |  |  |
| **Metric** | **Comparison** | **Δ Mean points** | **Wilcoxon *P*** | **Holm-adjusted *P*** | **Significance** |
| Recall | Bagging vs. KNN | 2.532 | 0.348 | 1 | ns |
|  | Bagging vs. MLP | -4.776 | 0.220 | 1 | ns |
|  | Bagging vs. Random Forest | 2.019 | 0.717 | 1 | ns |
|  | Bagging vs. SVM | 2.212 | 0.856 | 1 | ns |
|  | Bagging vs. XGBoost | -5.609 | 0.485 | 1 | ns |
|  | KNN vs. MLP | -7.308 | 0.088 | 1 | ns |
|  | KNN vs. Random Forest | -0.513 | 0.917 | 1 | ns |
|  | KNN vs. SVM | -0.321 | 0.711 | 1 | ns |
|  | KNN vs. XGBoost | -8.141 | 0.173 | 1 | ns |
|  | MLP vs. Random Forest | 6.795 | 0.073 | 1 | ns |
|  | MLP vs. SVM | 6.987 | 0.334 | 1 | ns |
|  | MLP vs. XGBoost | -0.833 | 0.793 | 1 | ns |
|  | Random Forest vs. SVM | 0.192 | 0.977 | 1 | ns |
|  | Random Forest vs. XGBoost | -7.628 | 0.170 | 1 | ns |
|  | SVM vs. XGBoost | -7.821 | 0.559 | 1 | ns |
|  |  |  |  |  |  |
|  |  |  |  |  |  |
| **Metric** | **Comparison** | **Δ Mean points** | **Wilcoxon *P*** | **Holm-adjusted *P*** | **Significance** |
| Specificity | Bagging vs. KNN | -3.237 | 0.312 | 1 | ns |
|  | Bagging vs. MLP | 4.038 | 0.222 | 1 | ns |
|  | Bagging vs. Random Forest | 2.692 | 0.530 | 1 | ns |
|  | Bagging vs. SVM | 3.205 | 0.736 | 1 | ns |
|  | Bagging vs. XGBoost | 11.410 | 0.055 | 0.744 | ns |
|  | KNN vs. MLP | 7.276 | 0.053 | 0.744 | ns |
|  | KNN vs. Random Forest | 5.929 | 0.111 | 1 | ns |
|  | KNN vs. SVM | 6.442 | 0.979 | 1 | ns |
|  | KNN vs. XGBoost | 14.647 | 0.016 | 0.246 | ns |
|  | MLP vs. Random Forest | -1.346 | 0.589 | 1 | ns |
|  | MLP vs. SVM | -0.833 | 0.243 | 1 | ns |
|  | MLP vs. XGBoost | 7.372 | 0.164 | 1 | ns |
|  | Random Forest vs. SVM | 0.513 | 0.410 | 1 | ns |
|  | Random Forest vs. XGBoost | 8.718 | 0.190 | 1 | ns |
|  | SVM vs. XGBoost | 8.205 | 0.157 | 1 | ns |
