# Supplementary material for: Diagnostic value of machine-learning using conventional magnetic resonance imaging markers for pediatric idiopathic intracranial hypertension: a retrospective study
Source: Pediatr Radiol. 2026 May 23;56(7):1516–35. doi: 10.1007/s00247-026-06638-7 (PMC13357526; doi:10.1007/s00247-026-06638-7)
Supplement: Supplementary file 8 — (DOCX 22.5 KB) [file 247_2026_6638_MOESM8_ESM.docx]

**Table 1** Most frequent and typical best-trial hyperparameters across classifier families

The table summarizes, for each classifier, the most frequently observed best-trial value and the typical interquartile-range interval across the best-performing Optuna trials. It provides an overview of how the repeated Bayesian optimization procedure configured random forest, support vector machine, multilayer perceptron, k-nearest neighbors, bagging, and extreme gradient boosting models. *IQR* interquartile range; *SVM* support vector machine; *MLP* multilayer perceptron; *KNN* k-nearest neighbors; *XGBoost* extreme gradient boosting; *RBF* radial basis function

| **Model / Parameter** | **Mode (most frequent)** | **Typical range (≈interquartile range across best trials)** | **Notes** |
| --- | --- | --- | --- |
| **Random forest** |  |  |  |
| n_estimators | 1000 | ≈800–1800 (200–2000 explored) | Large forests; best models generally use ≥800 trees. |
| max_depth | 4 | ≈37–56 (4–75 explored) | Trees are usually quite deep (≈40–60); a few runs pick shallow depth (4). |
| min_samples_split | 2 | ≈6–35 (2–46 explored) | Very small splits allowed, but typical best values are >5 to reduce overfitting. |
| min_samples_leaf | 2 | ≈2–10 (1–19 explored) | Leaves are small; ensembles plus depth act as main regularization. |
| max_features | log2 | {0.3, log2, 0.5, 0.7, …} | Mix of log2, sqrt/None-like and fractional (≈0.3–0.7) feature subsampling; no single dominant scheme. |
| criterion | entropy | {entropy, log_loss, gini} | Information-based criteria (entropy/log_loss) slightly preferred over Gini. |
| bootstrap | TRUE | {True, False} | Bootstrap slightly more common; both bagged and non-bagged forests appear. |
| **SVM** |  |  |  |
| kernel | rbf | {rbf, sigmoid, linear, poly} | RBF kernel dominates; sigmoid and linear appear; poly used only in a few runs. |
| C | — | ≈11.46–660.86 (6.8e-04–9998.69 explored) | Very wide C search; best trials mostly in moderate–high range (tens–hundreds). |
| gamma (mode) | scale | {scale, auto, float} | scale most frequent; auto and explicit float γ also chosen. |
| gamma (float) | — | ≈0.0011–0.1418 (1.1e-06–0.532 explored) | When gamma_mode="float", γ is typically in the 1e-3–1e-1 band. |
| degree (if poly) | — | ≈2–7 (2–7 explored) | Polynomial kernels are rare; degrees between 2 and 7 when used. |
| coef0 (if poly/sigmoid) | — | ≈−0.0411–3.64 (−4.74–4.81 explored) | Bias term only relevant for poly/sigmoid; values remain modest. |
| **MLP** |  |  |  |
| solver | lbfgs | {lbfgs, adam} | Both solvers appear; lbfgs more common in best models. |
| hidden_layer_sizes | (300, 150, 75) | Compact 1–3 layers, ≈50–300 units | Patterns like (300,150,75), (150,100), (100,100) are frequent; also smaller single-layer nets (50–150 units). |
| activation | tanh | {tanh, logistic, relu} | tanh and logistic are common; ReLU selected rarely. |
| alpha (L2) | — | ≈2.9e-05–0.0103 (1.8e-06–0.095 explored) | Weight decay mostly small–moderate; occasional heavier regularization. |
| max_iter | 1200 | ≈900–2400 (300–3000 explored) | Large iteration budgets; many runs likely converge before the cap. |
| learning_rate | adaptive | {adaptive, constant} | For Adam runs, adaptive schedule is clearly preferred to constant. |
| learning_rate_init | — | ≈1.0e-05–2.1e-04 (1.0e-06–0.003 explored) | Initial learning rates are small; typically 1e-5–2e-4. |
| batch_size | 64 | ≈32–64 (16–64 explored) | Small–medium mini-batches; 32–64 samples common. |
| early_stopping | FALSE | {False, True} | Early stopping sometimes used but not the dominant choice. |
| beta_1 | — | ≈0.5344–0.7948 (0.5035–0.797 explored) | Adam β₁ tuned below the usual 0.9; momentum ~0.53–0.79. |
| beta_2 | — | ≈0.8022–0.9077 (0.8–0.9095 explored) | Adam β₂ also reduced relative to 0.999; typically ~0.80–0.91. |
| **KNN** |  |  |  |
| n_neighbors | 3 | ≈3–8 (1–13 explored) | Small neighbourhoods; best models typically use 3–8 neighbours. |
| weights | distance | {distance, uniform} | Distance weighting slightly preferred to uniform. |
| metric | minkowski | {minkowski, chebyshev, manhattan, euclidean} | Best runs use several metrics; Minkowski, Chebyshev and Manhattan all appear often. |
| p | 2 | ≈2–4 (1–5 explored) | For Minkowski, exponents mostly between 2 and 4 (Euclidean-like). |
| leaf_size | 45 | ≈29–58 (15–89 explored) | Leaf size sits in a mid-range; effect likely weak on performance. |
| **Bagging** |  |  |  |
| base_estimator | knn | {knn, tree} | KNN base estimators more common (13/20) than decision trees (7/20). |
| n_estimators | 250 | ≈150–350 (50–500 explored) | Moderate-size ensembles; 150–350 base learners typical. |
| max_samples | — | ≈0.5361–0.9008 (0.3899–0.9867 explored) | Row subsampling generally between ~0.55 and 0.9 of training data. |
| max_features | — | ≈0.5485–0.7536 (0.3009–0.9861 explored) | Feature subsampling typically uses ~55–75% of features per base learner. |
| bootstrap | TRUE | {False, True} | Object-level bootstrap vs. no-bootstrap is roughly balanced. |
| bootstrap_features | FALSE | {False, True} | Feature bootstrapping off in most best runs. |
| max_depth (tree base) | 43 | ≈20–38 (2–43 explored) | For tree base-estimators only; trees moderately deep when used. |
| min_samples_leaf (tree base) | 7 | ≈6–8 (1–20 explored) | Tree leaves usually contain ~6–8 samples. |
| min_samples_split (tree base) | 34 | ≈29–34 (26–40 explored) | Splits require moderately large node sizes in tree base-estimators. |
| n_neighbors (knn base) | 1 | ≈1–4 (1–9 explored) | For KNN base-estimators, very small k (1–4) is preferred. |
| weights (knn base) | uniform | {uniform, distance} | For KNN base-estimators, uniform and distance weighting both used; slight tilt to uniform. |
| **XGBoost** |  |  |  |
| n_estimators | 900 | ≈900–2100 (300–3000 explored) | Many boosting rounds; ensembles with 900–2100 trees common. |
| max_depth | 13 | ≈8–17 (3–20 explored) | Trees relatively deep compared to typical shallow-gradient trees. |
| learning_rate | — | ≈0.0026–0.0665 (1.3e-04–0.2873 explored) | Learning rates in small–moderate range; smaller η when many trees are used. |
| subsample | — | ≈0.5272–0.7549 (0.3138–0.941 explored) | Row subsampling mostly around 0.5–0.75. |
| colsample_bytree | — | ≈0.4177–0.8137 (0.3004–0.9879 explored) | Column subsampling typically moderate (≈0.4–0.8). |
| min_child_weight | — | ≈0.0043–0.7815 (0.001–3.5 explored) | Often very small, allowing small leaves; occasionally larger for stronger regularization. |
| gamma | — | ≈2.08–4.65 (0.0775–8.11 explored) | Non-zero γ common; moderate–high split penalties. |
| reg_alpha / reg_lambda | — / — | α≈1.7e-07–0.0032; λ≈0.0065–0.7015 (full: α 1.2e-08–0.4113, λ 0.001–3.58) | Light–moderate L1 (α) and L2 (λ) regularization; α often near zero. |
| scale_pos_weight | — | ≈0.9985–1.84 (0.5607–2.96 explored) | Tuned around the imbalance ratio; typically near 1–2×. |
